# The Effects of Teleinterventions on Pediatric Weight Control: Systematic Review and Meta-Analysis of Randomized Controlled Trials

**DOI:** 10.2196/68688

**Published:** 2025-12-08

**Authors:** Cheng-Tai Wu, Jue-Chuan Ng, Yu-Tai Cheng, Ling-Yin Chang, Enoch Y Kang, Hsin-Hui Chiu, Ching-Feng Cheng

**Affiliations:** 1 Department of Pediatrics, Taipei Tzu Chi Hospital, Buddhist Tzu Chi Medical Foundation, 289 Jianguo Rd, Xindian, Taipei, 231, Taiwan, 886 2-66289779; 2 Institute of Health Behaviors and Community Sciences College of Public Health, National Taiwan University, Taipei, Taiwan; 3 Institute of Health Policy and Management College of Public Health, National Taiwan University, Taipei, Taiwan; 4 Department of Health Care Management, College of Health Technology, National Taipei University of Nursing and Health Sciences, Taipei, Taiwan; 5 Department of Pediatrics, Taipei Medical University School of Medicine, Taipei, Taiwan; 6 Institute of Biomedical Sciences, Academia Sinica, Taipei, Taiwan

**Keywords:** children, body weight, obese, nonpharmacological treatment, teleintervention, PRISMA

## Abstract

**Background:**

Childhood overweight or obesity has become one of the world’s most concerning health problems. Teleinterventions that deliver health information and behavioral strategies through telephone calls, websites, apps, newsletters, or emails can help manage children’s weight-related conditions.

**Objective:**

This study aimed to systematically evaluate the effects of teleinterventions versus nonteleinterventions on anthropometric outcomes in children with overweight or obesity.

**Methods:**

Randomized controlled trials investigating weight control using teleinterventions in children with overweight or obesity published in the Cochrane Library (including CENTRAL), Embase, PubMed, and Web of Science databases were selected. The outcomes included changes in BMI, BMI *z* score, body fat, and waist circumference. Two reviewers independently performed evidence selection, data extraction, and risk of bias evaluation. Data were pooled using a random-effects model. Results were presented as the mean difference (MD) with 95% CI.

**Results:**

A total of 26 randomized controlled trials involving 2866 children living with overweight or obesity met the eligibility criteria. The pooled results showed that teleinterventions significantly reduced the BMI *z* score between the fourth and sixth months (MD −0.15, 95% CI −0.23 to −0.08; *I*^2^=94%) and between the seventh and twelfth months (MD −0.19, 95% CI −0.34 to −0.03; *I*^2^=98%). Similarly, BMI (MD −2.48, 95% CI −4.15 to −0.82; *I*^2^=96%) and waist circumference (MD −0.59, 95% CI −1.05 to −0.14; *I*^2^=80%) were significantly reduced between the fourth and the sixth months but were nonsignificant between the seventh and twelfth months. Moreover, teleinterventions with family involvement or professional interaction between the fourth and the sixth months provided significant benefits, including reductions in BMI *z* score (MD −0.16, 95% CI −0.23 to −0.09; *I*^2^=96% and MD −0.13, 95% CI −0.20 to −0.05; *I*^2^=96%) and BMI (MD −2.50, 95% CI −4.32 to −0.69; *I*^2^=96% and MD −2.48, 95% CI −4.15 to −0.82; *I*^2^=96%). In addition, teleinterventions with family involvement could significantly reduce waist circumference between the fourth and sixth months (MD −0.75, 95% CI −1.25 to −0.25; *I*^2^=52%). Teleinterventions led to a significant reduction in waist circumference in children between the fourth and sixth months (MD −0.88, 95% CI −1.45 to −0.30; *I*^2^=75%). However, teleinterventions did not lead to a significant reduction in body fat among children or adolescents with overweight or obesity, even when family members were involved in the intervention.

**Conclusions:**

Teleinterventions, particularly when incorporating family engagement and structured professional interaction, yielded significant short- to medium-term improvements in weight control for children and adolescents living with overweight or obesity compared to nonteleinterventions. These findings highlight the promising role of telemedicine as a valuable modality for addressing the public health challenge of childhood obesity.

## Introduction

Childhood obesity has become a global health crisis in recent years, with alarming prevalence observed across geographic regions [1,2]. A comprehensive analysis of 2033 studies across 154 countries involving over 45 million individuals reported a global childhood and adolescent obesity prevalence of 8.5%, with regional rates ranging from 0.4% in Vanuatu to 28.4% in Puerto Rico, and showed a 1.5-fold increase between 2012 and 2023 compared to the prior decade [3]. Additionally, 22.2% of children and adolescents experienced excess weight, which correlated with regional income, the Human Development Index, and higher risks of depression and hypertension.

Addressing this multifactorial problem requires integrated strategies, as obesity increases the risk of systemic diseases such as type 2 diabetes, coronary artery disease, and stroke [1]. The management of childhood obesity involves both invasive and noninvasive approaches. Common invasive treatments include surgery and medication. Nevertheless, even children who receive medication or surgery still require health education regarding weight control and behavioral lifestyle counseling or behavioral change strategy interventions.

Telemedicine—including teleeducation, teleconsultation, and mobile apps—offers promising and cost-effective tools to enhance health care delivery [4]. Numerous studies have shown that teleinterventions can support weight management in children and adolescents by delivering health information or promoting behavioral changes through phone calls, websites, apps, newsletters, or emails [5-30]. These methods, which may also include remote visits and interactive messaging, complement in-person care and strengthen communication between health care providers, patients, and families [4,31]. Overall, teleinterventions provide a convenient, time-saving, and cost-effective approach to managing childhood obesity [4,31].

Most previous syntheses have explored teleinterventions for managing childhood obesity [32-36], some combined prevention and treatment [35,36], and few focused on anthropometric changes (ie, body fat reduction and waist circumference). Jahn Kassim et al [33] in their most recent synthesis emphasized behavioral effects rather than anthropometric outcomes. The largest targeted meta-analysis by Azevedo et al [32] included 19 randomized controlled trials (RCTs) with over 2000 participants and found that teleinterventions (excluding phone-based approaches) significantly reduced BMI and BMI *z* scores. However, methodological variability was present—4 trials delivered different intervention materials to control and experimental groups via teleinterventions [37-40] and 1 used offline active video games [41].

Despite promising findings, direct comparisons between teleinterventions and nonteleinterventions remain inconclusive. Further studies are warranted to explore effect modifiers such as age and baseline weight, which may influence outcomes [42]. Additionally, variations in implementation—such as levels of family involvement, professional interaction, and integration with in-person care—have yet to be comprehensively evaluated for their impact on effectiveness.

This study aimed to evaluate the impact of teleinterventions on anthropometric outcomes in children and adolescents living with overweight or obesity by synthesizing RCTs comparing teleintervention and nonteleintervention groups. It also sought to identify potential factors that may influence the effectiveness of teleinterventions in weight management.

## Methods

The protocol for this systematic review was prospectively registered on PROSPERO (CRD42022328874) before the formal search was initiated. The review process and reporting adhered to the Cochrane Handbook for Systematic Reviews of Interventions and the PRISMA (Preferred Reporting Items for Systematic Reviews and Meta-Analyses) statement [43,44].

### Eligibility Criteria

Studies meeting the following criteria were included: (1) RCT with individual or cluster randomization; (2) teleinterventions delivered via phone calls, text messages, websites, web apps, newsletters, emails, or other internet-based health education materials; (3) children and adolescents aged ≤18 years; (4) overweight or obesity was classified using international growth standards; and (5) control groups that received usual care, an in-person intervention, or no intervention at all. The exclusion criteria were as follows: (1) non-RCT designs, (2) conference reports or gray literature lacking complete data, (3) studies recruiting mixed populations that included adults or participants without overweight or obesity, (4) participants who were pregnant or had underlying or other systemic disease (eg, diabetes mellitus and Down syndrome), and (5) control groups that also received a teleintervention (Table S1 in Multimedia Appendix 1).

### Source and Search Strategy

The databases searched included the Cochrane Library (Cochrane database of Reviews and Cochrane CENTRAL−Central Register of Controlled Trials), Embase, PubMed, and Web of Science. A systematic search was conducted using keywords such as “online,” “children,” “overweight,” and “telemedicine,” combined with Boolean operators (OR within concepts; AND between concepts). No filters were applied for publication year, article type, full-text access, sex, age, or language. The search strategy was developed in the PubMed database and adapted for each database (see details in Table S2 in Multimedia Appendix 1). Two independent reviewers completed evidence selection, and the review team checked the final inclusions. The agreement between reviewers was 0.999 for title and abstract screening and 0.951 for full-text screening. The final search was completed on September 25, 2024.

### Data Extraction

Two researchers (CTW and JCN) extracted and verified trial data, including author name, publication year, inclusion period, participant age and sex, overweight or obesity status, and teleintervention design features (including family involvement, professional interaction, and integration with in-person visits). *Professional interaction* referred to direct engagement by health care professionals (eg, doctors, nurses, or other clinical professionals) to deliver information, monitor the condition, or ensure adherence—via phone calls, virtual visits, or other active guidance and progress assessment. Data on BMI, BMI *z* score, body fat, waist circumference, and adherence were collected, including means and SDs at baseline and follow-up. A standard month was defined as 4 weeks for consistency across studies using different time units. Outcomes were categorized by timing: within 3 months (before 13 weeks or 4 months), within 6 months (between 13 and 24 weeks or 4 and 6 months), and beyond 6 months (after 24 weeks or 6 months). For crossover trials, only first-period data were used to minimize carryover effects. We used the Data Estimation and Conversion for Meta-Analysis (DECoMA) tool to convert or estimate sample means and SDs when the original reports provided statistical measures other than means and SDs [45].

### Quality Evaluation

Two researchers (CTW and JCN) independently assessed the risk of bias using the Risk of Bias 2 (RoB 2) tool according to the Cochrane Handbook of Systematic Reviews of Intervention [42]. Disagreements were resolved through discussion with a third reviewer (EYK). The certainty of evidence was evaluated using the GRADE (Grading of Recommendations Assessment, Development and Evaluation) approach [42].

### Statistics

Meta-analysis was conducted using a random-effects model with the DerSimonian and Laird between-study variance estimator [46]. For continuous variables (BMI and BMI *z* score), results were calculated as mean difference (MD) to achieve consistent measures across studies. In contrast, body fat percentage and waist circumference were synthesized using standardized MD (SMD) due to variability in measurement units. Pooled effect sizes were presented with 95% CIs. Heterogeneity was assessed using the *I*^2^ statistic and its *P* value, with *I*^2^>50% indicating substantial heterogeneity, warranting cautious interpretation of the pooled results [47]. To explore potential sources of heterogeneity, subgroup analyses were performed based on trial-level characteristics, including patient age group and baseline body weight, as well as practical variations in teleintervention design, such as family involvement, professional interaction, and integration of in-person visits.

Publication bias was evaluated using funnel plots for pooled outcomes from more than 10 studies—specifically for the BMI *z* score at within 6-month and beyond 6-month follow-ups. All analyses were performed using Review Manager (RevMan; version 5.4; Cochrane Collaboration).

### Deviations From the Registered Protocol

In our original study protocol, we intended to perform subgroup analyses based on rural and urban differences; however, insufficient information to accurately classify study settings prevented us from conducting these analyses. Furthermore, while follow-up duration was not initially considered in the protocol, we recognized its importance during the study and subsequently incorporated meta-analyses stratified by follow-up time frames to better interpret the intervention effects.

## Results

### Overview

**Figure 1.**
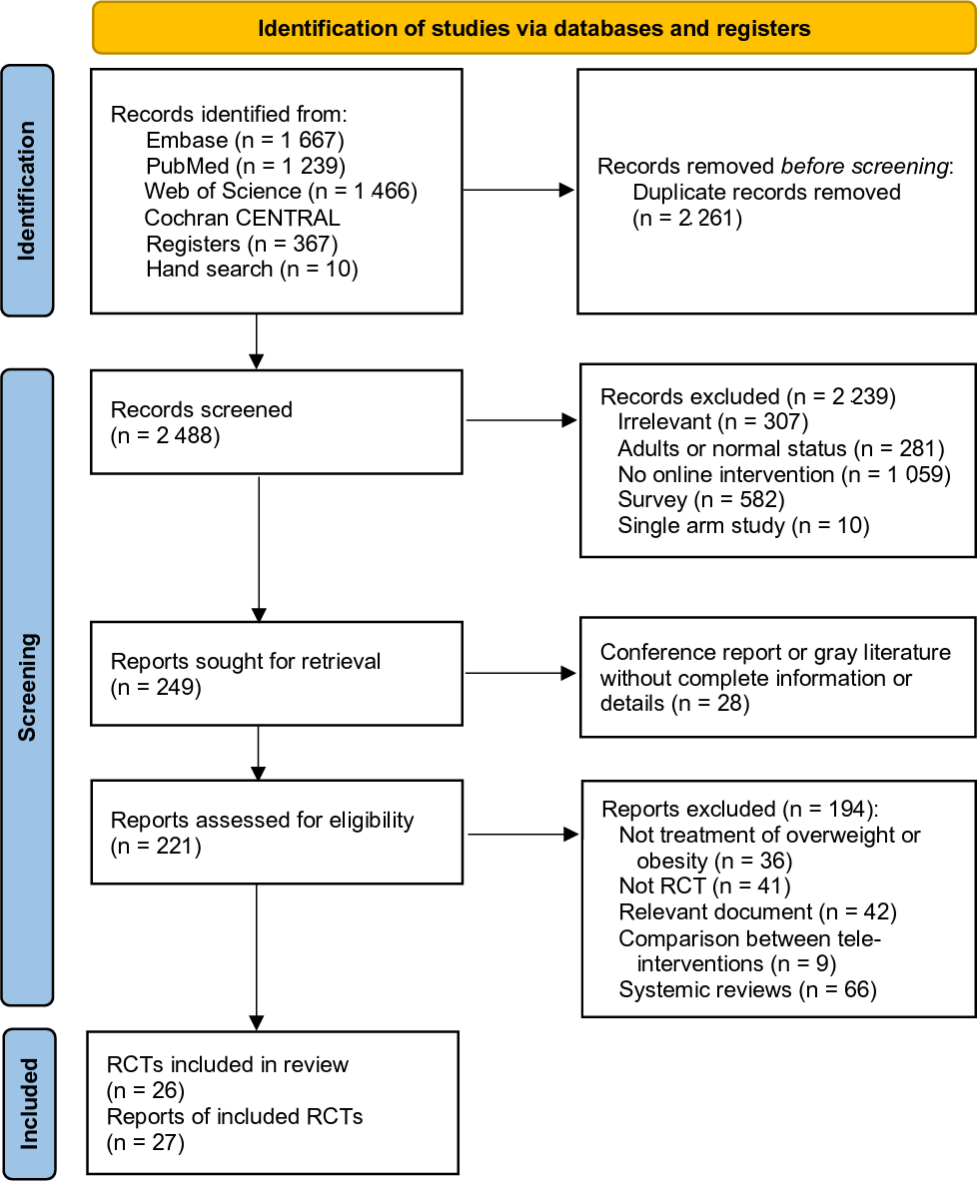
The flow of selection of evidence comparing pediatric weight control between teleinterventions and nonteleinterventions. Out of 4749 identified references, a total of 27 randomized controlled trial (RCT) reports were included.

Of a total of 4749 references, 4372 (92.1%) were identified from databases, 367 (7.7%) from trial registries, and 10 (0.2%) from manual searches. In total, 2261 (47.6%) duplicates and 2239 (47.1%) irrelevant references were excluded during title and abstract screening. Irrelevant references were excluded based on a clear mismatch with the eligibility criteria and were removed after screening titles and abstracts in accordance with PRISMA guidelines. A total of 149 (3.1%) references were further excluded due to (1) being conference reports or incomplete data, (2) having a preventive rather than therapeutic focus, (3) being limited to protocols or editor letters, despite being relevant, or (4) being systematic reviews (Table S3 in Multimedia Appendix 1). Ultimately, 27 (0.6%) references from 26 RCTs met the eligibility criteria (Figure 1).

### Characteristics

A total of 26 RCTs with 2866 children (aged 5.4 to 16.8 years) with overweight or obesity were included in our study [5-30]. Definitions of pediatric overweight and obesity in the studies aligned with the rules by the World Health Organization [48]. Most trials (17/26, 65%) focused on obese participants, with others (7/26, 27%) including mixed overweight or obese groups, and very few (1/26, 4%) trials included participants at or above grade 5 according to the World Health Organization cutoffs. Regarding intervention design, 42% (11/26) of trials involved family participation in at least one group, 65% (17/26) of trials included professional interaction in at least one group, and 35% (9/26) of trials combined teleinterventions with in-person training in at least one group. Sex distribution (1288/2866, 44.9% boys and 1303/2866, 45.5% girls) was reported in most studies. The interventions in these studies were classified into a teleintervention group if they delivered health information or strategically treated children via phone, web, web application, web newsletter, or email. These RCTs were hospital-based or health professional–led initiatives and not government programs (Table 1). A total of 85% (22/26) of RCTs had a low risk of bias, 12% (3/26) of RCTs had some concerns, and only 4% (1/26) of trials had a high risk of bias (Table S4 in Multimedia Appendix 1).

Based on available data, there were no significant differences in BMI [5,8,13,15,16,18,19,21,24,25,28,29], body fat [5,6,10,18,20,22,23,25], and waist circumference between pairs of groups at baseline (Figures S1-S3 in Multimedia Appendix 1) [6-14,17,19,20,22-24,26-29]; while the baseline BMI *z* score was significantly higher in the teleintervention groups (MD 0.07, 95% CI 0.00-0.13; *I*^2^=74%) compared with the control groups (Figure S4 in Multimedia Appendix 1). No significant difference was found in the adherence rate between pairs of groups, with a Peto odds ratio of 1.2 (95% CI 0.92-1.56; *I*^2^=72%; Figure S5 in Multimedia Appendix 1). The number of RCTs reporting the BMI *z* score, BMI, body fat, and waist circumference varied across time frames. The BMI *z* score was most commonly (12/26, 46%) reported beyond 6 months, while the BMI was consistently (6/26, 23%) reported within 3 and 6 months but less often beyond that. Body fat and waist circumference were reported in fewer studies (10/26, 38%), especially within 3 months (≤12 weeks; Table S5 in Multimedia Appendix 1).

**Table 1.** Characteristics of randomized controlled trials in this synthesis.

Trial and weight condition		Intervention strategy				Sex, n	Mean age
		Tele^a^	FI^b^	PI^c^	IPT^d^		
Abraham et al [5] (2015), obesity (≥95th percentile)							
	Group 1	–	–	+	–	Male: 10; female: 6	14.3^e^
	Group 2	–	+	+	–	Male: 10; female: 6	14.9^e^
	Group 3	+	–	+	+	Male: 9; female: 7	14.1^e^
Ahmad et al [6] (2018), obesity^f^ (≥85th percentile)							
	Group 1	–	–	–	–	Male: 29; female: 38	9.6
	Group 2	+	+	+	+	Male: 27; female: 40	9.6
Bohlin et al [7] (2017), obesity^f^							
	Group 1	–	+	+	–	Male: 14; female: 4	9.3
	Group 2	+	+	+	+	Male: 10; female: 9	9.8
Delli Bovi [8] (2021), obesity, (≥95th percentile)							
	Group 1	–	–	+	–	Male: 25; female: 24	10.4
	Group 2	+	+	+	+	Male: 24; female: 30	9.7
Currie et al [9] (2018), obesity (≥95th percentile)							
	Group 1	–	–	+	–	Both: male: 28; female: 36	14.4
	Group 2	+	–	+	+	(Both: male: 28; female: 36)^g^	(14.4)
Davis et al [10] (2012), obesity^f^ (≥85th percentile)							
	Group 1	–	–	+	–	Male: 17; female: 13	15.6
	Group 2	+	–	+	–	Male: 7; female: 16	15.8
de Niet et al [11] (2012), obesity^f^							
	Group 1	–	+	+	–	Male: 23; female: 45	9.8
	Group 2	+	+	+	+	Male: 28; female: 45	10.0
Estabrooks et al [12] (2009), obesity^f^ (≥85th percentile)							
	Group 1	–	+	–	–	Male: 39; female: 11	11.0
	Group 2	–	+	+	–	Male: 58; female: 27	10.6
	Group 3	+	+	+	–	Male: 59; female: 26	10.7
Fleischman et al [13] (2016), obesity (≥95th percentile)							
	Group 1	–	+	+	–	Both: male: 9; female: 31	14.3
	Group 2	+	+	+	+	(Both: male: 9; female: 31)^g^	(14.3)
Johansson et al [14] (2020), obesity							
	Group 1	–	+	+	–	Male: 7; female: 6	9.8
	Group 2	+	+	+	+	Male: 6; female: 9	8.4
Lee et al [15] (2017), obesity (≥95th percentile)							
	Group 1	–	+	+	–	Male: 0; female: 0	15.3
	Group 2	+	+	+	+	Male: 0; female: 0	13.4
Likhitweerawong et al [16] (2002), obesity (≥95th percentile)							
	Group 1	–	–	+	–	Male: 24; female: 11	12.8
	Group 2	+	–	+	+	Male: 24; female: 11	13.1
Love-Osborne et al [17] (2014), obesity (≥95th percentile)							
	Group 1	–	+	+	–	Male: 45; female: 38	16
	Group 2	+	+	+	+	Male: 34; female: 48	15.7
Mohammed Nawi et al [18] (2015), obesity^f^ (BMI ≥25)							
	Group 1	–	–	–	–	Male: 30; female: 20	16.0
	Group 2	+	–	–	–	Male: 25; female: 22	16.0
Nguyen et al [19] (2013), obesity^f^ (BMI z: 1‐2.5)							
	Group	–	+	+	–	Male and female: 78	N/A^h^
	Group	+	+	+	–	Male and female: 73	N/A
Patrick et al [20] (2013), obesity^f^ (≥85th percentile)							
	Group 1	–	–	–	–	Male: 7; female: 18	14.5
	Group 2	+	–	+	–	Male: 10; female: 16	14.1
	Group 3	+	+	+	+	Male: 12; female: 14	14.3
	Group 4	+	–	+	–	Male: 8; female: 16	14.3
Ptomey et al [21] (2021), obesity^f^ (≥85th percentile)							
	Group 1	–	+	+	–	Male: 20; female: 16	16.3
	Group 2	+	+	+	+	Male: 15; female: 24	15.6
	Group 3	+	+	+	+	Male: 17; female: 18	16.7
Staiano et al [22] (2018), overweight^i^ (≥85th percentile)							
	Group 1	–	–	–	–	Male and female: 23	N/A
	Group 2	+	+	+	+	Male and female: 23	N/A
Stasinaki et al [23] (2021), obesity^f^ (≥90th percentile)							
	Group 1	–	–	+	–	Male and female: 13	13.7^e^
	Group 2	+	–	+	+	Male and female: 18	12.6^e^
Taveras et al [24] (2015), obesity (≥95th percentile)							
	Group 1	–	–	+	–	Male: 100; female: 84	9.8
	Group 2	+	+	+	+	Male: 101; female: 93	9.8
	Group 3	+	+	+	+	Male: 91; female: 80	9.8
Tugault-Lafleur [30] (2023), overweight^j^							
	Group 1	–	+	–	–	Male: 47; female: 60	13.1
	Group 2	+				Male: 57; female: 50	12.8
Tsiros et al [25] (2008), obesity							
	Group 1	–	–	–	–	Male and female: 22	14.6
	Group 2	+	+	+	+	Male and female: 25	14.6
Vermeiren et al [26] (2021), obesity							
	Group 1	–	+	+	–	Male: 37; female: 49	13.0
	Group 2	+	+	+	+	Male: 33; female: 57	13.2
Vidmar et al [27] (2021), obesity (≥95th percentile)							
	Group 1	–	–	+	–	Male: 3; female: 12	16.38
	Group 2	–	–	+	–	Male: 6; female: 13	16.16
	Group 3	+	–	+	–	Male: 5; female: 11	16.8
Wake et al [28] (2013), obesity							
	Group 1	–	–	+	–	Male: 33; female: 23	7.4
	Group 2	+	–	+	+	Male: 31; female: 31	7.2
Wald et al [29] (2017), mixed (≥85th percentile)							
	Group 1	–	+	+	–	Male: 14; female: 21	5.4
	Group 2	+	+	+	+	Male: 20; female: 18	5.5

a Tele: teleintervention.

b FI: family involvement.

c PI: professional interaction.

d IPT: in-person training.

e These values were reported as medians.

f In these studies, the majority of cases had obesity and some cases had overweight.

g In these studies, “both” indicates that the authors did not break down the numbers of participants in the control and intervention groups, but only reported combined numbers. Parentheses have been added to indicate that these values should not be double counted.

h N/A: not available.

i In these studies, the majority of cases had overweight and some cases had obesity.

j This study defined overweight as grade 5 or above in the age- and sex-specific World Health Organization cutoffs.

### Synthesis Results

#### BMI z Score Analysis

**Figure 2.**
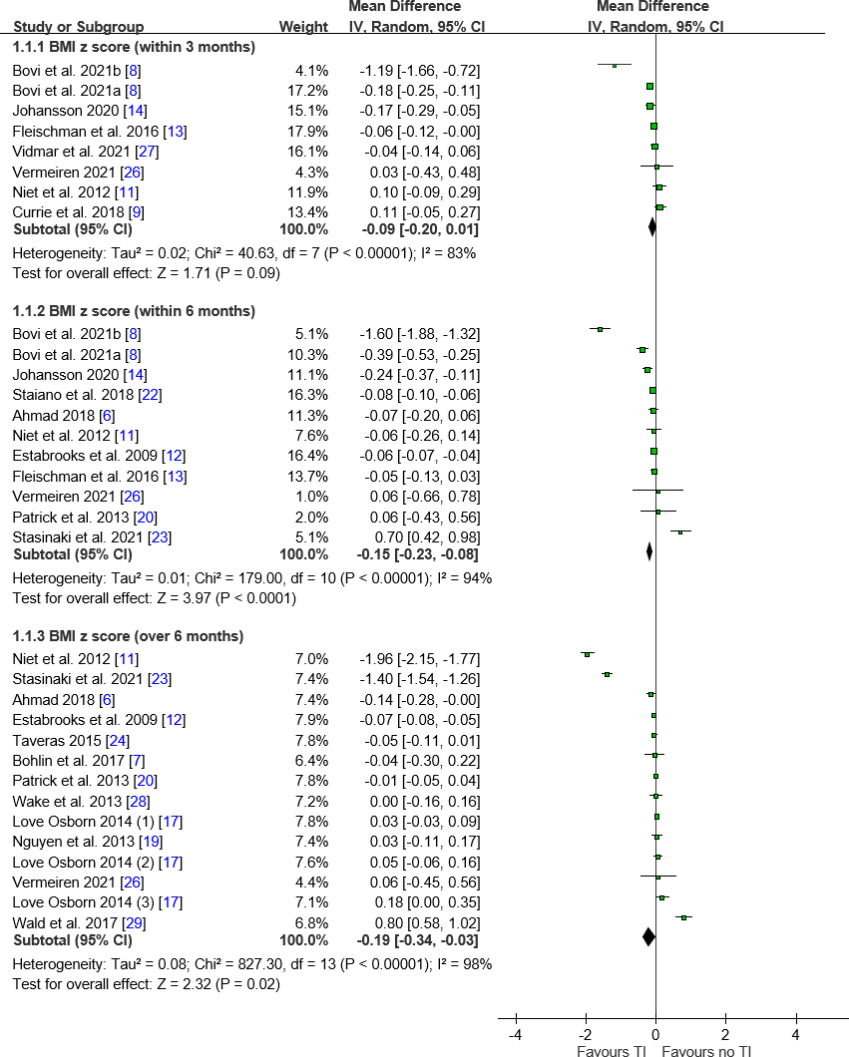
Forest plot of differences in the BMI *z* score. Generally, favorable trends were noted toward teleinterventions in the BMI *z* score. IV: inverse variance; MD: mean difference; TI: teleintervention.

A BMI *z* score analysis showed significant reductions in the teleintervention groups at both within 6-month (MD −0.15, 95% CI −0.23 to −0.08; *I*²=94%) and beyond 6-month (MD −0.19, 95% CI −0.34 to −0.03; *I*²=98%) follow-ups but not in the within 3-month follow-up period (Figure 2). Subgroup analyses showed that children aged below 12 years experienced significant BMI *z* score reductions at the within-3-month and within-6-month intervals but not beyond. Individuals with obesity showed improvements at the within-6-month follow-up and beyond. Professional interaction yielded significant effects at both time intervals, while family involvement was beneficial within 3 months and within 6 months. The in-person intervention yielded a significant effect only beyond the 6-month follow-up (Table 2). Despite high heterogeneity, these results suggest targeted components may enhance teleintervention effectiveness.

**Table 2.** Summary of subgroup analysis by children or teens, overweight or obesity, and program designs.

Outcome or subgroup	Within 3 months			Within 6 months			Beyond 6 months		
RCT^a^ (cases)	ES^b^ (95% CI)	*I* ^2^	RCTs (cases)	ES (95% CI)	*I* ^2^	RCTs (cases)	ES (95% CI)	*I* ^2^
BMI *z* score^c^									
Children (aged 0 to 12 y)	3 (272)	−0.24 (−0.46 to −0.01)	88%	6 (621)	−0.23 (−0.31 to −0.15)	96%	7 (1176)	−0.21 (−0.50 to 0.08)	99%
Teens	4 (289)	−0.03 (−0.09 to 0.04)	27%	4 (255)	0.21 (−0.25 to 0.66)	88%	5 (558)	−0.16 (−0.45 to 0.14)	98%
Overweight	N/A^e^	N/A	N/A	1 (45)	−0.08 (−0.10 to −0.06)	N/A	2 (116)	0.48 (−0.13 to 1.10)	95%
Obesity	7 (561)	−0.09 (−0.20 to 0.01)	83%	9 (831)	−0.18 (−0.35 to −0.01)	95%	11 (1618)	−0.29 (−0.46 to −0.13)	99%
Professionals interaction	7 (561)	−0.09 (−0.20 to 0.01)	83%	10 (850)	−0.13 (−0.20 to −0.05)	96%	12 (1708)	−0.19 (−0.34 to −0.03)	98%
No professional interaction	N/A	N/A	N/A	1 (50)	0.00 (−0.04 to 0.04)	N/A	1 (50)	0.00 (−0.05 to 0.05)	N/A
Family involvement	5 (447)	−0.15 (−0.29 to −0.01)	85%	9 (797)	−0.16 (−0.23 to −0.09)	96%	10 (1540)	−0.11 (−0.25 to 0.04)	98%
No family involvement	2 (114)	0.02 (−0.12 to 0.17)	58%	2 (103)	0.36 (−0.28 to 1.00)	95%	3 (218)	−0.45 (−1.34 to 0.44)	99%
In-person intervention	6 (462)	−0.11 (−0.28 to 0.06)	85%	9 (734)	−0.13 (−0.26 to 0.01)	96%	11 (1649)	−0.20 (−0.38 to −0.03)	99%
BMI (kg/m^2^)									
Children (aged 0 to 12 y)	1 (103)	−1.23 (−1.72 to −0.74)	N/A	1 (103)	−5.64 (−8.88 to −2.39]	93%	3 (709)	−0.15 (−0.72 to 0.43)	0%
Teens	5 (302)	−0.26 (−0.87 to 0.36)	44%	5 (354)	−1.14 (−2.10 to −0.19)	83%	1 (107)	0.60 (−1.03 to 2.23)	N/A
Overweight	N/A	N/A	N/A	N/A	N/A	N/A	1 (73)	0.20 (−0.71 to 1.11)	N/A
Obesity	6 (405)	−0.64 (−1.24 to −0.04)	67%	6 (457)	−2.48 (−4.15 to −0.82)	96%	3 (743)	−0.21 (−0.88 to 0.47)	0%
Professional interaction	5 (308)	−0.65 (−1.30 to −0.00)	73%	6 (457)	−2.48 (−4.15 to −0.82)	96%	4 (816)	−0.06 (−0.61 to 0.48)	0%
No professional interaction	1 (97)	−0.50 (−2.37 to 1.37)	N/A	N/A	N/A	N/A	N/A	N/A	N/A
Family involvement	3 (190)	−0.66 (−1.35 to 0.03)	80%	5 (409)	−2.50 (−4.32 to −0.69)	96%	3 (698)	−0.02 (−0.60 to 0.56)	0%
No family involvement	3 (215)	−0.56 (−2.07 to 0.95)	47%	1 (48)	−2.35 (−4.49 to −0.21)	N/A	1 (118)	−0.40 (−1.93 1.13)	N/A
In-person intervention	4 (211)	−0.31 (−0.94 to 0.32)	64%	4 (256)	−2.52 (−5.18 to 0.14)	97%	4 (816)	−0.06 (−0.61 to 0.48)	5%
Body fat^d^									
Children (aged 0 to 12 y)	N/A	N/A	N/A	2 (179)	−0.14 (−0.72 to 0.43)	66%	2 (252)	−0.15 (−0.39 to 0.10)	0%
Teens	2 (145)	−0.15 (−0.60 to 0.30)	39%	5 (310)	−0.34 (−0.85 to 0.18)	74%	2 (126)	−0.23 −(2.02 to 1.57)	93%
Overweight	N/A	N/A	N/A	1 (45)	−0.49 (−1.09 to 0.10)	N/A	N/A	N/A	N/A
Obesity	2 (145)	−0.15 (−0.60 to 0.30)	39%	6 (444)	−0.23 (−0.64 to 0.18)	72%	4 (378)	−0.22 (−0.79 to 0.34)	84%
Professional interaction	1 (48)	−0.44 (−1.05 to 0.16)	N/A	7 (463)	−0.28 (−0.66 to 0.09)	71%	4 (352)	−0.22 (−0.77 to 0.33)	82%
No professional interaction	1 (97)	0.03 (−0.37 to 0.43)	N/A	1 (50)	−0.50 (−1.07 to 0.06)	N/A	1 (50)	−1.35 (−1.97 to −0.73)	N/A
Family involvement	N/A	N/A	N/A	5 (362)	−0.18 (−0.55 to 0.20)	63%	2 (184)	−0.13 (−0.42 to 0.16)	0%
No family involvement	2 (145)	−0.15 (−0.60 to 0.30)	39%	3 (151)	−0.34 (−1.16 to 0.47)	81%	3 (218)	−0.38 (−1.52 to 0.77)	92%
In-person intervention	N/A	N/A	N/A	5 (348)	−0.00 (−0.38 to 0.38)	61%	4 (340)	−0.06 (−0.34 to 0.22)	33%
Waist circumference									
Children (aged 0 to 12 y)	1 (103)	−0.86 (−1.65 to −0.08)	N/A	2 (237)	−0.88 (−1.45 to −0.30)	75%	2 (252)	−0.29 (−0.54 to −0.04)	0%
Teens	4 (255)	−0.16 (−0.48 to 0.15)	34%	5 (241)	−0.42 (−1.12 to 0.29)	83%	2 (135)	0.41 (−0.33 to 1.15)	66%
Overweight	N/A	N/A	N/A	N/A	N/A	N/A	N/A	N/A	N/A
Obesity	5 (358)	−0.40 (−0.79 to −0.01)	69%	7 (478)	−0.59 (−1.05 to −0.14)	80%	4 (387)	−0.01 (−0.40 to 0.39)	70%
Professional interaction	4 (261)	−0.47 (−0.96 to 0.02)	73%	7 (478)	−0.59 (−1.05 to −0.14)	80%	4 (387)	−0.01 (−0.40 to 0.39)	70%
No professional interaction	1 (97)	−0.14 (−0.54 to 0.26)	N/A	N/A	N/A	N/A	N/A	N/A	N/A
Family involvement	2 (143)	−0.75 (−1.25 to −0.25)	52%	5 (399)	−0.77 (−1.24 to −0.30)	77%	2 (241)	−0.15 (−0.65 to 0.35)	74%
No family involvement	3 (215)	−0.07 (−0.40 to 0.25)	28%	2 (79)	0.06 (−1.44 to 1.56)	90%	2 (146)	0.29 (−0.73 to 1.32)	82%
In-person intervention	3 (164)	−0.23 (−0.74 to 0.27)	62%	5 (277)	−0.59 (−1.39 to 0.22)	88%	4 (387)	−0.01 (−0.40 to 0.39)	70%

a RCT: randomized controlled trial.

b ES: effect size.

c Mean difference.

d Standardized mean difference.

e N/A: not available.

#### BMI Analysis

**Figure 3.**
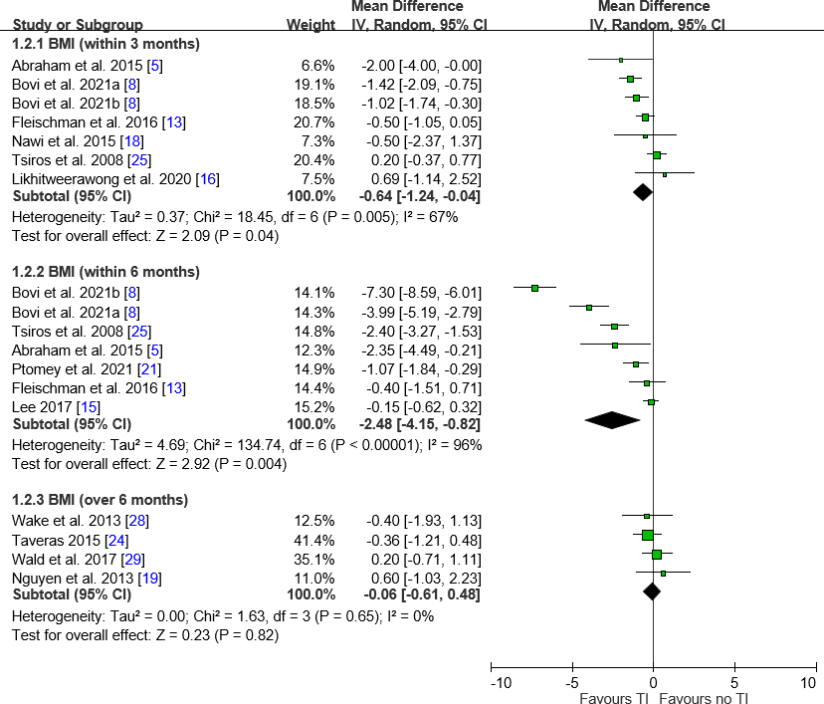
Forest plot of differences in BMI. Generally, favorable trends were noted toward teleinterventions in BMI. IV: inverse variance; MD: mean difference; TI: teleintervention.

BMI analysis showed significant reductions in the teleintervention groups at both within 3-month (MD −0.64, 95% CI −1.24 to −0.04; *I*²=67%) and within 6-month (MD −2.48, 95% CI −4.15 to −0.82; *I*²=96%) follow-ups but no significant changes beyond 6 months (Figure 3). Subgroup analyses of children aged below 12 years showed significant BMI reductions within 3 months and within 6 months, while teens showed improvement within 6 months. Individuals with obesity showed improvements at within 3-month and within 6-month intervals. Professional interaction was effective within 3 and 6 months, and family involvement was associated with a significant reduction at the within-6-month follow-up (Table 2).

#### Body Fat Analysis

**Figure 4.**
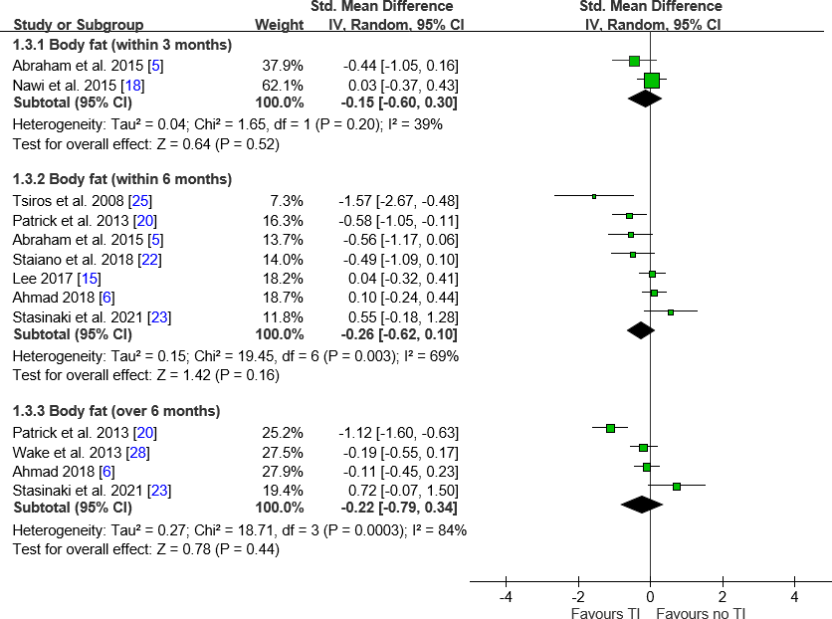
Forest plot of differences in body fat. No significant differences were noted between teleinterventions and nonteleinterventions in body fat. IV: inverse variance; MD: mean difference; TI: teleintervention.

Body fat analysis showed no significant overall difference between the teleintervention and control groups (Figure 4). Subgroup analyses also revealed few significant findings due to limited data availability (Table 2). The only significant result was in the “no professional interaction” subgroup beyond 6 months (MD −1.35, 95% CI −1.97 to −0.73). All other subgroups—including by age, weight status, professional or family involvement, and delivery method—showed no significant changes at any time intervals.

#### Waist Circumference Analysis

**Figure 5.**
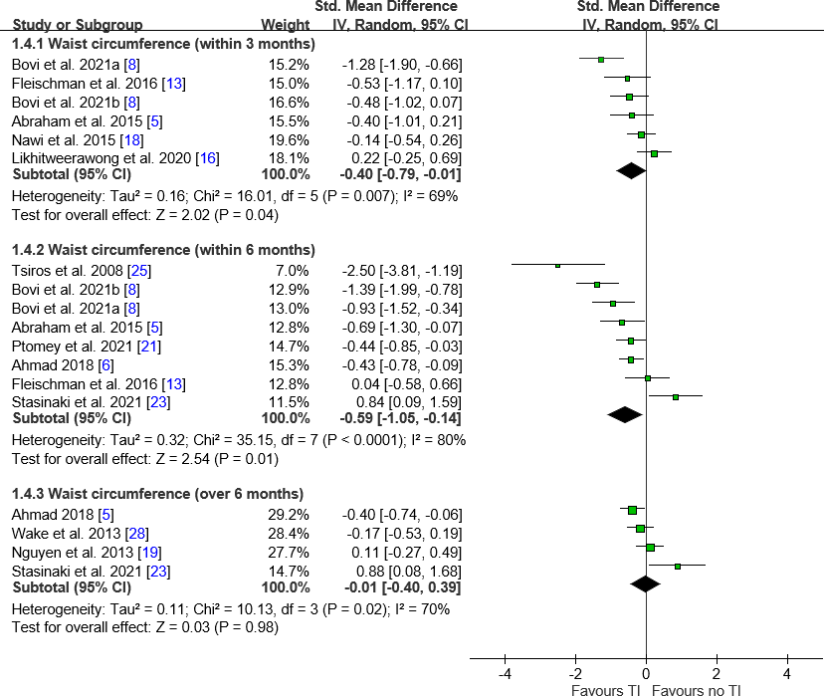
Forest plot of differences in waist circumference. Generally, favorable trends were noted toward teleinterventions in waist circumference. IV: inverse variance; MD: mean difference; TI: teleintervention.

Waist circumference was significantly lower in the teleintervention group at both the within-3-month (SMD −0.40, 95% CI −0.79 to −0.01; *I*²=69%) and within-6-month (SMD −0.59, 95% CI −1.05 to −0.14; *I*²=80%) intervals (Figure 5). Subgroup analyses showed significant reductions in the groups of children at all time intervals, including a modest effect beyond 6 months (MD −0.29, 95% CI −0.54 to −0.04; *I*²=0%). Participants with obesity and those with family involvement showed significant reductions within 3 and 6 months. Professional interaction was associated with reductions in waist circumference within 6 months (Table 2).

### Publication Bias

**Figure 6.**
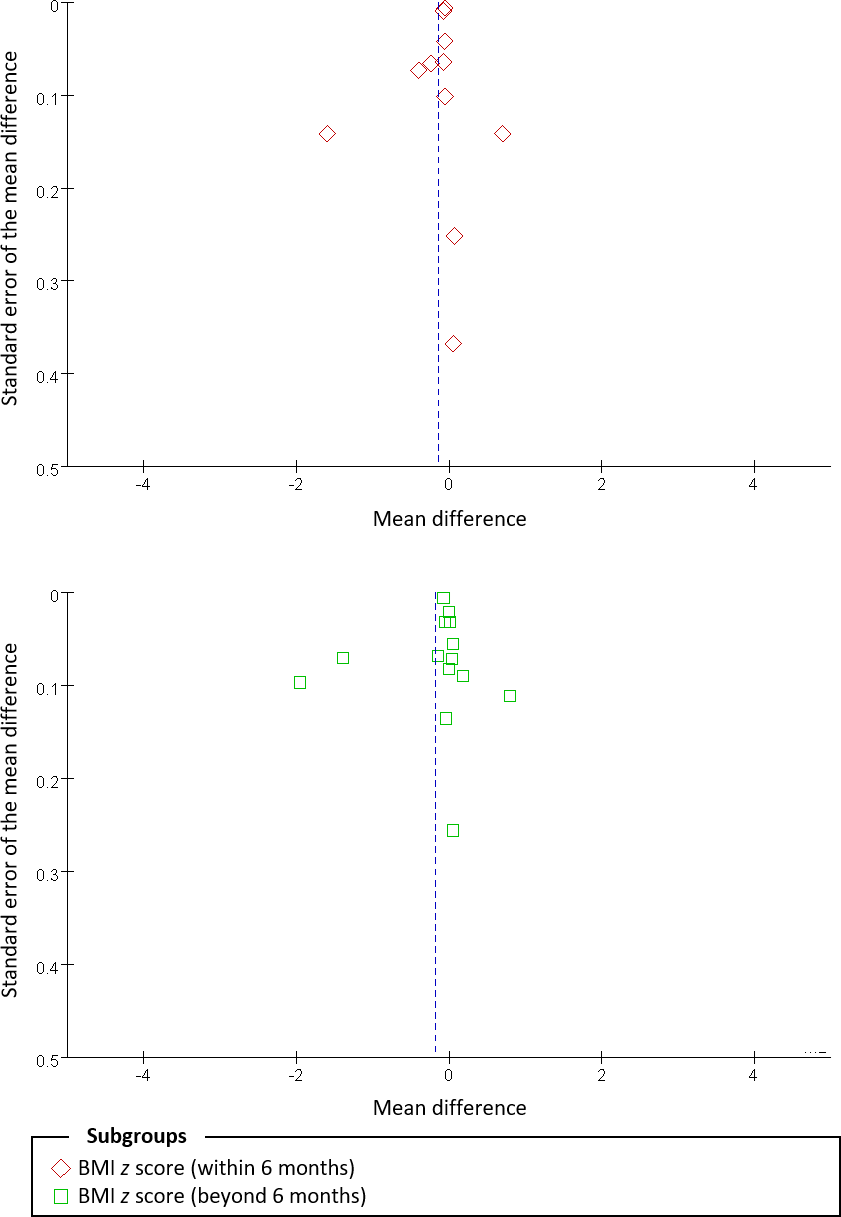
Funnel plots of differences in the BMI *z* score. No obvious asymmetric pattern could be observed.

Since only outcomes of the within-6-month and beyond-6-month BMI *z* score were based on data from more than 10 studies, funnel plots were generated for these analyses, and the outcomes were not seriously biased by small-study effects, as no obvious asymmetry was observed in the funnel plots (Figure 6). Besides, further statistical analysis was not conducted because of the nonasymmetric plots.

## Discussion

### Principal Findings

This synthesis showed that teleinterventions could significantly reduce BMI *z* scores, with effects lasting beyond 6 months. A similar trend was observed for BMI, with favorable benefits mainly appearing within 6 months but becoming nonsignificant beyond 6 months. This might be due to (1) the novelty of the intervention gradually wearing off, weakening children’s motivation or (2) delayed improvements in control group children. Overall, teleinterventions appeared effective for pediatric obesity management, aligning with prior research [49]. Although the direction of findings did not change based on studies with a low risk of bias, the certainty of most findings remained low to moderate (Table S5 in Multimedia Appendix 1), partly due to potential effect modifiers such as population characteristics and intervention strategies. Subgroup analyses suggested that teleinterventions might reduce BMI *z* scores in children within 6 months but not in adolescents.

### Comparison to Prior Work

Teleinterventions show promise for weight management, potentially by improving health literacy and promoting behavior change—even without explicit behavior-focused strategies [49]. However, pediatric overweight and obesity are multifactorial conditions shaped by diet, physical activity, sedentary behaviors, and family dynamics [49]. Parents play a key role through their own health behaviors and literacy [49]. Our findings support this, showing significant improvements in BMI *z* score and waist circumference when family involvement is integrated into teleinterventions. This highlights the importance of addressing social and environmental influences, particularly parental involvement.

Parents influence children’s eating habits through modeling, food choices, and feeding practices [50,51]. However, engaging parents in regular in-person programs is often challenging. Teleinterventions offer a flexible alternative, enabling tracking of physical activity and diet while fostering engagement through digital platforms [52].

In contrast, adolescents—who value autonomy and are less influenced by parents [52]—respond less consistently to teleinterventions. One possible reason is the lack of peer involvement in teleinterventions. Peer influence is well known to affect physical activity and eating behavior [53]. Adolescents tend to mirror the behaviors of their peers, such as eating or exercising together. Interventions based on expectancy value and social cognitive theories suggest that peer support enhances engagement in physical activity [53]. However, most studies in this synthesis did not incorporate peer-focused strategies, potentially limiting the effectiveness of teleinterventions for adolescents living with overweight or obesity.

### Strengths and Limitations

This meta-analysis has several strengths. It followed rigorous Cochrane and PRISMA guidelines, ensuring transparency and reproducibility. A comprehensive, unrestricted search strategy across multiple databases minimized the risk of missing relevant studies. By including only RCTs, the review offers high-quality evidence on the effectiveness of teleinterventions. Independent, duplicate data extraction and quality assessments enhanced the reliability of findings. Subgroup analyses by age, baseline weight, and teleintervention design components provide insight into potential effect modifiers. Additionally, assessing publication bias for the primary outcome strengthens the robustness of the conclusions.

However, the study also has notable limitations. First, while teleintervention is a delivery method, its practical applications vary widely. Although subgroup analyses identified key components such as family involvement and professional interaction, further studies are needed to determine the most effective design for pediatric weight control. Second, few studies reported long-term outcomes for BMI, body fat, and waist circumference, limiting the certainty of longer-term effects. Third, the lack of comprehensive data on physical activity, eating behaviors, intervention adherence, and screen time hindered a deeper exploration of these variables’ influence. Finally, there was no consistent threshold defining the proportion of teleintervention sessions across studies, making it difficult to compare intervention intensity and potentially affecting outcome consistency.

### Future Directions 

Future studies should address current limitations and explore teleinterventions’ potential in managing childhood body weight. Larger, well-designed RCTs with extended follow-up are needed to confirm long-term effects on various anthropometric outcomes. Standardizing teleintervention components and delivery methods would reduce heterogeneity and improve comparability. Researchers should also examine the mechanisms of action and the optimal combinations of delivery modes (eg, remote visits, text messages, and mobile apps). Additionally, studies should investigate how individual factors—such as socioeconomic status and family dynamics—influence effectiveness. Qualitative studies could provide insights into participant experiences, while cost-effectiveness analyses are essential for assessing the sustainability and scalability of these interventions in real-world settings.

### Conclusions

Our study supported teleinterventions as effective strategies for improving weight control outcomes—specifically BMI *z* score (within and beyond 6 months), BMI, and waist circumference (within 6 months)—in children and adolescents living with overweight or obesity. Subgroup analyses showed greater benefits in younger children (less than 12 years), those with obesity, and interventions with professional interaction or family involvement.

Although teleinterventions showed promise, the variability in intervention design highlighted the need for standardized, well-designed studies. Future researchers should identify optimal delivery components, assess long-term effects on multiple anthropometric outcomes, and examine individual and contextual factors influencing success. A clearer understanding of implementation strategies will enhance the impact and scalability of teleinterventions in combating childhood obesity.

## Supplementary material

10.2196/68688 Multimedia Appendix 1 Details of systematic search, evidence selection, baseline comparability, risk of bias evaluation, and certainty of evidence.

10.2196/68688 Checklist 1 PRISMA checklist.

## Abbreviations

DECoMA: Data Estimation and Conversion for Meta-Analysis
MD: mean difference
PRISMA: Preferred Reporting Items for Systematic Reviews and Meta-Analyses
RCT: randomized controlled trial
SMD: standardized mean difference
WHO: World Health Organization
