# Supplementary material for: The Effects of Teleinterventions on Pediatric Weight Control: Systematic Review and Meta-Analysis of Randomized Controlled Trials
Source: J Med Internet Res. 2025 Dec 8;27:e68688. doi: 10.2196/68688 (PMC12685287; doi:10.2196/68688)
Supplement: Multimedia Appendix 1 [file jmir-v27-e68688-s001.docx]

## MULTIMEDIA APPENDIX 1

# The Effects of Teleinterventions on Pediatric Weight Control: Systematic Review and Meta-Analysis of Randomized Controlled Trials

**Cheng-Tai Wu,** M.D.^1,*^, **Jue-Chuan Ng,** M.D.^1^, **Yu-Tai Cheng,** M.D.^1^, **Ling-Yin Chang,** Ph.D.^2,3^, **Enoch Kang,** M.A.^2,3,4,*^, **Hsin-Hui Chiu,** M.D., Ph.D.^1,5,6^, **Ching-Feng Cheng,** M.D., Ph.D.^1,7,*^

1. Department of Pediatrics, Taipei Tzu Chi Hospital, Buddhist Tzu Chi Medical Foundation, New Taipei City 231, Taiwan

2. Institute of Health Behaviors and Community Sciences, College of Public Health, National Taiwan University

3. Institute of Health Policy and Management, College of Public Health, National Taiwan University, Taipei, Taiwan

4. Department of Health Care Management, College of Health Technology, National Taipei University of Nursing and Health Sciences, Taipei, Taiwan

5. Department of Pediatrics, School of Medicine, College of Medicine, Taipei Medical University, Taipei, Taiwan

6. Department of Pediatrics, National Taiwan University Children's Hospital, Taipei, Taiwan

7. Institute of Biomedical Sciences, Academia Sinica, Taipei 115, TaiwanDepartment of Emergency Medicine, Taipei Medical University Hospital, Taipei, Taiwan

* these authors contributed equally

Corresponding Author: Hsin-Hui Chiu, MD, PhD

**Content**

**Supplementary Table 1** Eligibility criteria

**Supplementary Table 2** Database and search strategy

**Supplementary Table 3** Excluded references with reasons in the step of full-text retrieval and review

**Supplementary Table 4** Risk of bias (Cochrane risk-of-bias tool 2, RoB 2)

**Supplementary Table 5** Summary of finding of the Grading of Recommendations Assessment, Development and Evaluation

**Supplementary Figure 1** Forest plot of body mass index at baseline

**Supplementary Figure 2** Forest plot of body fat at baseline

**Supplementary Figure 3** Forest plot of waist circumference at baseline

**Supplementary Figure 4** Forest plot of BMI z-score at baseline

**Supplementary Table 1** Eligibility criteria

| **Article type** | |  |
| --- | --- | --- |
| *The inclusion criteria for article type:*  1. Randomized controlled trial  2. Randomized crossover trial  3. Cluster randomized controlled trial | *The respective exclusion criteria:*  1. Review or expert opinion  2. Case report  3. Case series  4. Cross-sectional study (e.g. survey)  5. Case control  6. Cohort study  7. Single-arm trial  8. Non-randomized trial  9. Systematic review  10. Meta-analysis |  |
| **Language** | |  |
| *The inclusion criteria for language:*  All languages | *The respective exclusion criteria for language:*  None |  |
| **Publication time** | |  |
| *The inclusion criteria for time:*  All publication time point | *The respective exclusion criteria for time:*  None |  |
| **Target population** | |  |
| *The inclusion criteria for population:*  1. Children with overweight  2. Children with obesity | *The respective exclusion criteria for population:*  1. Adults that cannot separated from children  2. Children with normal body status  . (non-overweight)  3. Children with overweight or obesity due to specific  . disease |  |
| **Treatment** | |  |
| *The inclusion criteria for treatment:*  1. health education via phone calls  2. health education via text message  3. health education via websites  4. health education via web apps  5. health education via newsletters  6. health education via email  7. health education via internet-based way | *The respective exclusion criteria for treatment:*  1. tele-intervention without information about health  2. physical health education  3. physical visiting  4. local game-based intervention |  |
| **Comparison** | | |
| *The inclusion criteria for comparison:*  1. waiting  2. physical health education  3. physical visiting  4. local game-based intervention | | *The respective exclusion criteria for comparison:*  1. health education via phone calls  2. health education via text message  3. health education via websites  4. health education via web apps  5. health education via newsletters  6. health education via email  7. health education via internet-based way |

**Supplementary Table 2** Database and search strategy

**Primary search strategy:**

#01 online

#02 web-based

#03 smartphone

#04 smart-phone

#05 cellphone

#06 cell-phone

#07 cellular phone

#08 mobile phone

#09 phone

#10 tele*

#11 remote

#12 #01 OR #02 OR #03 OR #04 OR #05 OR #06 OR #07 OR #08 OR #09 OR #010 OR #11

#13 Obesity

#14 Obese

#15 overweight

#16 weight

#17 adiposity

#18 #13 OR #14 OR #15 OR #16 OR #17

#19 pediatric

#20 pediatrics

#21 paediatric

#22 paediatrics

#23 child

#24 children

#25 kid

#26 kids

#27 teenage

#28 teenager

#29 teenagers

#30 adolescent

#31 adolescents

#32 #19 OR #20 OR #21 OR #22 OR #23 OR #24 OR #25 OR #26 OR #27 OR #28 OR #29 OR #30 OR #31

#33 #12 AND #18 AND #32

**Cochrane CENTRAL (i = 367):**

online OR web-based OR smartphone OR smart-phone OR cellphone OR cell-phone OR cellular phone OR mobile phone OR phone OR tele* OR remote in Title Abstract Keyword AND obesity OR obese OR overweight OR weight OR adiposity in Record Title AND pediatric OR pediatrics OR paediatric OR paediatrics OR child OR children OR kid OR kids OR teenage OR teenager OR teenagers OR adolescent OR adolescents in Record Title - (Word variations have been searched)

**Embase (i = 1667):**

('pediatric'/mj OR 'pediatrics'/mj OR 'paediatric'/mj OR paediatrics:ti OR 'child'/mj OR 'child':ti OR 'children':ti OR kid:ti OR kids:ti OR teenage:ti OR 'adolescent'/mj OR 'adolescent' OR 'teenager' OR teenagers:ti OR 'adolescents'/mj) AND ('obesity'/mj OR 'adipose tissue hyperplasia':ti OR 'adipositas':ti OR 'adiposity':ti OR 'alimentary obesity':ti OR 'body weight, excess':ti OR 'corpulency':ti OR 'fat overload syndrome':ti OR 'nutritional obesity':ti OR 'obesitas':ti OR 'obesity':ti OR 'overweight':ti OR obese:ti) AND ('online'/exp OR 'web based' OR 'smartphone'/exp OR 'smart phone' OR 'smartphone' OR 'smartphones' OR 'mobile phone'/exp OR 'cell phone' OR 'cell phones' OR 'cellphone' OR 'cellphones' OR 'cellular telephone' OR 'mobile phone' OR 'mobile telephone' OR 'phone call'/exp OR phone OR tele*:ti,ab OR remote:ti,ab)

**PubMed (i = 1239):**

(online[tiab] OR web-based[tiab] OR smartphone[tiab] OR smart-phone[tiab] OR cellphone[tiab] OR cell-phone[tiab] OR cellular phone[tiab] OR mobile phone[tiab] OR phone[tiab] OR tele*[tiab] OR remote[tiab]) AND (obesity[ti] OR obese[ti] OR overweight[ti] OR weight[ti] OR adiposity[ti]) AND (pediatric[ti] OR pediatrics[ti] OR paediatric[ti] OR paediatrics[ti] OR child[ti] OR children[ti] OR kid[ti] OR kids[ti] OR teenage[ti] OR teenager[ti] OR teenagers[ti] OR adolescent[ti] OR adolescents[ti])

**Web of Science (i = 1466):**

TS=(online OR web-based OR smartphone OR smart-phone OR cellphone OR cell-phone OR cellular phone OR mobile phone OR phone OR tele* OR remote) AND TI=(obesity OR obese OR overweight OR weight OR adiposity) AND TI=(pediatric OR pediatrics OR paediatric OR paediatrics OR child OR children OR kid OR kids OR teenage OR teenager OR teenagers OR adolescent OR adolescents)

**Supplementary Table 3** Excluded references with reasons in the step of full-text retrieval and review

**Intervention for prevention but not treatment of overweight or obesity (i = 36)**

1. Armstrong B, Trude ACB, Johnson C, et al. CHAMP: A cluster randomized-control trial to prevent obesity in child care centers. *Contemp Clin Trials*. Nov 2019;86:105849. doi:10.1016/j.cct.2019.105849

2. Arthurs N, Browne S, Boardman R, et al. The BigO application: Usability and engagement among adolescents and children with obesity. Conference Abstract. *Obesity Reviews*. 2020;21(SUPPL 1)doi:10.1111/obr.13118

3. Askie LM, Espinoza D, Martin A, et al. Interventions commenced by early infancy to prevent childhood obesity—The EPOCH Collaboration: An individual participant data prospective meta-analysis of four randomized controlled trials. Article. *Pediatric Obesity*. 2020;15(6)doi:10.1111/ijpo.12618

4. Austin SB, Spadano-Gasbarro JL, Greaney ML, et al. Effect of the planet health intervention on eating disorder symptoms in Massachusetts middle schools, 2005-2008. Article. *Preventing chronic disease*. 2012;9:E171; quiz E171. doi:10.5888/pcd9.120111

5. Brambilla P, Bedogni G, Buongiovanni C, et al. "Mi voglio bene": a pediatrician-based randomized controlled trial for the prevention of obesity in Italian preschool children. *Ital J Pediatr*. Aug 17 2010;36:55. doi:10.1186/1824-7288-36-55

6. Byrd-Bredbenner C, Martin-Biggers J, Koenings M, Quick V, Hongu N, Worobey J. HomeStyles, A Web-Based Childhood Obesity Prevention Program for Families With Preschool Children: Protocol for a Randomized Controlled Trial. *JMIR Res Protoc*. Apr 25 2017;6(4):e73. doi:10.2196/resprot.7544

7. Byrd-Bredbenner C, Santiago E, Eck KM, et al. HomeStyles-2: Randomized controlled trial protocol for a web-based obesity prevention program for families with children in middle childhood. *Contemp Clin Trials*. Nov 30 2021:106644. doi:10.1016/j.cct.2021.106644

8. Cruz TH, Davis SM, FitzGerald CA, Canaca GF, Keane PC. Engagement, recruitment, and retention in a trans-community, randomized controlled trial for the prevention of obesity in rural American Indian and Hispanic children. *J Prim Prev*. Jun 2014;35(3):135-49. doi:10.1007/s10935-014-0340-9

9. French SA, Gerlach AF, Mitchell NR, Hannan PJ, Welsh EM. Household obesity prevention: Take actiona group-randomized trial. Article. *Obesity*. 2011;19(10):2082-2088. doi:10.1038/oby.2010.328

10. Haines J, McDonald J, O'Brien A, et al. Healthy Habits, Happy Homes: randomized trial to improve household routines for obesity prevention among preschool-aged children. *JAMA Pediatr*. Nov 2013;167(11):1072-9. doi:10.1001/jamapediatrics.2013.2356

11. Hammersley ML, Jones RA, Okely AD. Time2bHealthy - An online childhood obesity prevention program for preschool-aged children: A randomised controlled trial protocol. *Contemp Clin Trials*. Oct 2017;61:73-80. doi:10.1016/j.cct.2017.07.022

12. Hammersley ML, Okely AD, Batterham MJ, Jones RA. An Internet-Based Childhood Obesity Prevention Program (Time2bHealthy) for Parents of Preschool-Aged Children: Randomized Controlled Trial. *J Med Internet Res*. Feb 8 2019;21(2):e11964. doi:10.2196/11964

13. Horodynski MA, Olson B, Baker S, et al. Healthy babies through infant-centered feeding protocol: an intervention targeting early childhood obesity in vulnerable populations. Article. *BMC public health*. 2011;11:868.

14. Knowlden A, Sharma M. One-Year Efficacy Testing of Enabling Mothers to Prevent Pediatric Obesity Through Web-Based Education and Reciprocal Determinism (EMPOWER) Randomized Control Trial. *Health Educ Behav*. Feb 2016;43(1):94-106. doi:10.1177/1090198115596737

15. Knowlden AP, Conrad E. Two-Year Outcomes of the Enabling Mothers to Prevent Pediatric Obesity Through Web-Based Education and Reciprocal Determinism (EMPOWER) Randomized Control Trial. *Health Educ Behav*. Apr 2018;45(2):262-276. doi:10.1177/1090198117732604

16. Knowlden AP, Sharma M. Process evaluation of the Enabling Mothers toPrevent Pediatric Obesity Through Web-Based Learning and Reciprocal Determinism (EMPOWER) randomized control trial. *Health Promot Pract*. Sep 2014;15(5):685-94. doi:10.1177/1524839914523431

17. Knowlden AP, Sharma M, Cottrell RR, Wilson BR, Johnson ML. Impact evaluation of Enabling Mothers to Prevent Pediatric Obesity through Web-Based Education and Reciprocal Determinism (EMPOWER) Randomized Control Trial. *Health Educ Behav*. Apr 2015;42(2):171-84. doi:10.1177/1090198114547816

18. Liu Z, Gao P, Gao AY, et al. Effectiveness of a Multifaceted Intervention for Prevention of Obesity in Primary School Children in China: A Cluster Randomized Clinical Trial. *JAMA Pediatr*. Nov 8 2021:e214375. doi:10.1001/jamapediatrics.2021.4375

19. Liu Z, Wu Y, Niu WY, et al. A school-based, multi-faceted health promotion programme to prevent obesity among children: protocol of a cluster-randomised controlled trial (the DECIDE-Children study). *BMJ Open*. Nov 2 2019;9(11):e027902. doi:10.1136/bmjopen-2018-027902

20. Lombard C, Deeks A, Jolley D, Ball K, Teede H. A low intensity, community based lifestyle programme to prevent weight gain in women with young children: cluster randomised controlled trial. *Bmj*. Jul 13 2010;341:c3215. doi:10.1136/bmj.c3215

21. Lubans DR, Smith JJ, Plotnikoff RC, et al. Assessing the sustained impact of a school-based obesity prevention program for adolescent boys: the ATLAS cluster randomized controlled trial. *Int J Behav Nutr Phys Act*. Aug 20 2016;13:92. doi:10.1186/s12966-016-0420-8

22. Markert J, Herget S, Petroff D, et al. Telephone-based adiposity prevention for families with overweight children (T.A.F.F.-Study): one year outcome of a randomized, controlled trial. *Int J Environ Res Public Health*. Oct 3 2014;11(10):10327-44. doi:10.3390/ijerph111010327

23. Nyström CD, Sandin S, Henriksson P, et al. Mobile-based intervention intended to stop obesity in preschool-aged children: the MINISTOP randomized controlled trial. *Am J Clin Nutr*. Jun 2017;105(6):1327-1335. doi:10.3945/ajcn.116.150995

24. Raat H, Struijk MK, Remmers T, et al. Primary prevention of overweight in preschool children, the BeeBOFT study (breastfeeding, breakfast daily, outside playing, few sweet drinks, less TV viewing): design of a cluster randomized controlled trial. *BMC public health*. Oct 19 2013;13doi:10.1186/1471-2458-13-974

25. Robinson TN. Reducing children's television viewing to prevent obesity - A randomized controlled trial. *JAMA-journal of the american medical association*. Oct 27 1999;282(16):1561-1567. doi:10.1001/jama.282.16.1561

26. Ruiter ELM, Fransen GAJ, Molleman GRM, van der Velden K, Engels R. The effectiveness of a web-based Dutch parenting program to prevent overweight in children 9-13 years of age: study protocol for a two-armed cluster randomized controlled trial. *BMC public health*. Feb 14 2015;15doi:10.1186/s12889-015-1394-1

27. Sherwood NE, Levy RL, Langer SL, et al. Healthy Homes/Healthy Kids: a randomized trial of a pediatric primary care-based obesity prevention intervention for at-risk 5-10 year olds. *Contemp Clin Trials*. Sep 2013;36(1):228-43. doi:10.1016/j.cct.2013.06.017

28. Simon C, Schweitzer B, Oujaa M, et al. Successful overweight prevention in adolescents by increasing physical activity: a 4-year randomized controlled intervention. *International journal of obesity*. Oct 2008;32(10):1489-1498. doi:10.1038/ijo.2008.99

29. Taveras EM, Gortmaker SL, Hohman KH, et al. Randomized controlled trial to improve primary care to prevent and manage childhood obesity: the High Five for Kids study. *Arch Pediatr Adolesc Med*. Aug 2011;165(8):714-22. doi:10.1001/archpediatrics.2011.44

30. van Grieken A, Vlasblom E, Wang L, et al. Personalized Web-Based Advice in Combination With Well-Child Visits to Prevent Overweight in Young Children: Cluster Randomized Controlled Trial. *J Med Internet Res*. Jul 27 2017;19(7):e268. doi:10.2196/jmir.7115

31. Veldhuis L, Struijk MK, Kroeze W, et al. 'Be active, eat right', evaluation of an overweight prevention protocol among 5-year-old children: design of a cluster randomised controlled trial. *BMC public health*. Jun 8 2009;9doi:10.1186/1471-2458-9-177

32. Verbestel V, De Coen V, Van Winckel M, Huybrechts I, Maes L, De Bourdeaudhuij I. Prevention of overweight in children younger than 2 years old: a pilot cluster-randomized controlled trial. *PUBLIC HEALTH NUTRITION*. JUN 2014;17(6):1384-1392. doi:10.1017/S1368980013001353

33. Weintraub DL, Tirumalai EC, Haydel KF, Fujimoto M, Fulton JE, Robinson TN. Team sports for overweight children - The Stanford sports to prevent obesity randomized trial (SPORT). *Archives of pediatrics & adolescent medicine*. Mar 2008;162(3):232-237. doi:10.1001/archpediatrics.2007.43

34. Wen LM, Rissel C, Baur LA, et al. A 3-Arm randomised controlled trial of Communicating Healthy Beginnings Advice by Telephone (CHAT) to mothers with infants to prevent childhood obesity. Article. *BMC public health*. 2017;17(1):79. doi:10.1186/s12889-016-4005-x

35. Wen LM, Rissel C, Xu HL, et al. Linking two randomised controlled trials for Healthy Beginnings (c): optimising early obesity prevention programs for children under 3years. *BMC public health*. Jun 13 2019;19doi:10.1186/s12889-019-7058-9

36. Wilken LR, Novotny R, Fialkowski MK, et al. Children's Healthy Living (CHL) Program for remote underserved minority populations in the Pacific region: rationale and design of a community randomized trial to prevent early childhood obesity. *BMC public health*. Oct 9 2013;13doi:10.1186/1471-2458-13-944

**Conference report or gray literature without details (i = 28)**

37. Adams b. Dose-response effects of an online and in-person family intervention on physical activity in children who are overweight. *Annals of behavioral medicine*. May 2020;54:S26-S26.

38. Alff F, Markert J, Zschaler S, Gausche R, Kiess W, Blüher S. Reasons for (non)participating in a telephone-based intervention program for families with overweight children. *PLoS One*. 2012;7(4):e34580. doi:10.1371/journal.pone.0034580

39. Bean MK, Mendoza A, Farthing S, Smith D, Adams EL, Caccavale L. Transitioning an adolescent obesity treatment from in-person to remote delivery: lessons learned from the teens plus trial. *Annals of behavioral medicine*. Apr 2021;55:S543-S543.

40. Bean MK, Thornton LM, Jeffers AJ, Gow RW, Mazzeo SE. Impact of motivational interviewing on engagement in a parent-exclusive paediatric obesity intervention: randomized controlled trial of NOURISH plus MI. *Pediatric obesity*. Apr 2019;14(4)doi:10.1111/ijpo.12484

41. Bovi APD, Cesari GM, Rocco MC, et al. Healthy lifestyle management of pediatric obesity with a hybrid system of customized mobile technology: The pediafit pilot project. Article. *Nutrients*. 2021;13(2):1-15. doi:10.3390/nu13020631

42. Browne S, Doyle G, Kechadi T, et al. Mobile health (mHealth) applications with children in treatment for obesity: A randomised feasibility study. Conference Abstract. *Proceedings of the Nutrition Society*. 2020;79(OCE2)doi:10.1017/S0029665120003420

43. Chew CSE, Davis C, Lim JKE, et al. Use of a Mobile Lifestyle Intervention App as an Early Intervention for Adolescents With Obesity: Single-Cohort Study. *J Med Internet Res*. Sep 28 2021;23(9):e20520. doi:10.2196/20520

44. Davis AM, James RL, Boles RE, Goetz JR, Belmont J, Malone B. The use of TeleMedicine in the treatment of paediatric obesity: feasibility and acceptability. *Matern Child Nutr*. Jan 2011;7(1):71-9. doi:10.1111/j.1740-8709.2010.00248.x

45. Davis AM, Sampilo M, Gallagher KS, et al. Treating rural paediatric obesity through telemedicine vs. telephone: Outcomes from a cluster randomized controlled trial. *Journal of telemedicine and telecare*. Mar 2016;22(2):86-95. doi:10.1177/1357633X15586642

46. Davis AM, Sampilo M, Gallagher KS, Landrum Y, Malone B. Treating rural pediatric obesity through telemedicine: outcomes from a small randomized controlled trial. *J Pediatr Psychol*. Oct 2013;38(9):932-43. doi:10.1093/jpepsy/jst005

47. DuBose KD, Currie J, Collier D, Raedeke TD, Kemble CD. The Effect of a Telephone-Based Physical Activity Intervention in Obese Adolescents. *Medicine and science in sports and exercise*. May 2014;46(5):170-170. doi:10.1249/01.mss.0000493688.62146.8d

48. Felix S, Ramalho S, Conceicao E, Saint-Maurice PF, Silva D, Mansilha HF. Satisfaction with Apolo-Teens Online Program for Overweight/Obese Adolescents. *Annals of nutrition and metabolism*. 2018;73(4):318-319.

49. Félix S, Ramalho S, Ribeiro E, Conceição E. Mothers and adolescents' experience regarding an online intervention for adolescents with overweight and obesity: A pilot study. Conference Abstract. *Obesity Reviews*. 2020;21(SUPPL 1)doi:10.1111/obr.13118

50. Felix S, Ramalho S, Ribeiro E, et al. Experiences of parent-adolescent dyads regarding a Facebook-based intervention to improve overweight/obesity treatment in adolescents: A qualitative study. *Applied psychology-health and well being*. doi:10.1111/aphw.12294

51. Jones A, Mann K, Cutler L, et al. Targeting parental recognition and understanding of childhood overweight to improve child weight outcomes: The impact of the MapMe intervention at 12 months follow-up. Conference Abstract. *Obesity Facts*. 2017;10:11-12. doi:10.1159/000468958

52. Kornman KP, Shrewsbury VA, Chou AC, et al. Electronic therapeutic contact for adolescent weight management: the Loozit study. *Telemed J E Health*. Jul-Aug 2010;16(6):678-85. doi:10.1089/tmj.2009.0180

53. L'Allemand D, Shih CH, Heldt K, et al. Design and interim evaluation of a smartphone app for overweight adolescents using a behavioural health intervention platform. Conference Abstract. *Obesity Reviews*. 2018;19:102. doi:10.1111/(ISSN)1467-789X

54. Lang L. Text Messaging May Help Children to Fight Off Obesity. Article. *Gastroenterology*. 2009;136(1):7-8. doi:10.1053/j.gastro.2008.11.047

55. Markert J, Herget S, Vogel M, Gausche R, Hilbert A, Blüher S. After care treatment approach for adolescent obesity via telephone counseling following an obesity treatment program-study concept. Conference Abstract. *Hormone Research in Paediatrics*. 2012;78:279. doi:10.1159/000343184

56. Nguyen B, Shrewsbury V, Lau C, et al. Adolescent and parent views of an adolescent weight management program: Lessons from the Loozit® randomised controlled trial. Conference Abstract. *Obesity Research and Clinical Practice*. 2012;6:56. doi:10.1016/j.orcp.2012.08.114

57. Nguyen B, Shrewsbury V, O'Connor J, et al. Two-year outcomes of an extended adolescent weight-loss maintenance intervention involving novel additional therapeutic contact: The Loozit® randomised controlled trial. Conference Abstract. *Obesity Research and Clinical Practice*. 2012;6:38-39. doi:10.1016/j.orcp.2012.08.079

58. Nguyen B, Shrewsbury V, O'Connor J, et al. Two-year outcomes of an extended adolescent weight-loss maintenance intervention involving novel additional therapeutic contact: The Loozit®R randomised controlled trial. Conference Abstract. *Obesity Facts*. 2012;5:188-189. doi:10.1159/000258190

59. Parra-Medina D, Mojica CM, Parma DL, Rubalcava L, Ramos AI. Preliminary results from an obesity management intervention in a rural pediatric practice: The NEST study. Conference Abstract. *Obesity*. 2011;19:S110. doi:10.1038/oby.2011.226

60. Ramalho S, Silva D, Mansilha H, Saint Maurice P, Conceição E. APOLO-Teens, a Web-based intervention for adolescents with overweight/obesity seeking treatment: An effectiveness study. Conference Abstract. *Obesity Facts*. 2019;12:74. doi:10.1159/000489691

61. Tabak RG, Tate DF, Stevens J, Siega-Riz AM, Ward DS. Family ties to health study: A randomized intervention to improve vegetable intake in children. Conference Abstract. *Obesity*. 2011;19:S109. doi:10.1038/oby.2011.226

62. Werk LN, Hossain J, Martinez A, et al. Extending obesity care beyond the office doors using telemedicine health coaches. A pilot test randomized trial. Conference Abstract. *Pediatrics*. 2019;144(2)doi:10.1542/peds.144.2-MeetingAbstract.219

63. Wu TJ, Holt NM, Dalton W, Maphis L, Schetzina K. A child weight management program targeting parents in primary care: insights from plan for healthy living phone follow-ups. *Annals of behavioral medicine*. Apr 2012;43:s194-s194.

64. Wyse R, Wolfenden L, Campbell E, et al. Increasing fruit and vegetable consumption in 3- 5 year old children: Results from a cluster randomised controlled trial of a telephone-based parent intervention, hunter region, NSW, Australia. Conference Abstract. *Obesity Reviews*. 2011;12:68. doi:10.1111/j.1467-789X.2011.00889.x

**Relevant documents (letter to the editor or protocol, i = 39)**

65. Beeken RJ, Croker H, Morris S, et al. Study protocol for the 10 Top Tips (10TT) trial: randomised controlled trial of habit-based advice for weight control in general practice. *BMC public health.* 2012;12:667.

66. Brennan L, Walkley J, Fraser SF, Greenway K, Wilks R. Motivational interviewing and cognitive behaviour therapy in the treatment of adolescent overweight and obesity: study design and methodology. *Contemp Clin Trials.* 2008;29(3):359-375.

67. De Assis MAA, Rolland-Cachera MF, De Vasconcelos FDAG, et al. Overweight and thinness in 7-9 year old children from Florianópolis, Southern Brazil: A comparison with a French study using a similar protocol. *Revista de Nutricao.* 2006;19(3):299-308.

68. Deehan EC, Colin-Ramirez E, Triador L, et al. Efficacy of metformin and fermentable fiber combination therapy in adolescents with severe obesity and insulin resistance: study protocol for a double-blind randomized controlled trial. *Trials.* 2021;22(1):148.

69. Falconer C, Park M, Skow A, et al. Scoping the impact of the national child measurement programme feedback on the child obesity pathway: study protocol. *BMC Public Health.* 2012;12:783.

70. Hill JL, Heelan KA, Bartee RT, et al. A Type III Hybrid Effectiveness-Implementation Pilot Trial Testing Dissemination and Implementation Strategies for a Pediatric Weight Management Intervention: The Nebraska Childhood Obesity Research Demonstration Project. *Child Obes.* 2021;17(S1):S70-s78.

71. Karmali S, Ng V, Battram D, et al. Coaching and/or education intervention for parents with overweight/obesity and their children: study protocol of a single-centre randomized controlled trial. *BMC Public Health.* 2019;19(1):345.

72. Kim JE, Jang HB, Lee HJ, et al. Rationale and design of theory-based nutritional strategy to promote healthy dietary habits in severe obese adolescents: Study protocol for ICAAN (intervention for childhood and adolescent obesity via activity and nutrition) project. *FASEB Journal.* 2017;31(1).

73. Lim C, Rutledge L, Sandridge S, King K, Jefferson D, Tucker T. Design, Implementation, and Examination of a Remote Patient Monitoring System for Pediatric Obesity: Protocol for an Open Trial Pilot Study. *JMIR Res Protoc.* 2021;10(7):e29858.

74. Liu S, Marques IG, Perdew MA, et al. Family-based, healthy living intervention for children with overweight and obesity and their families: a 'real world' trial protocol using a randomised wait list control design. *BMJ Open.* 2019;9(10):e027183.

75. Mâsse LC, Vlaar J, Macdonald J, et al. Aim2Be mHealth intervention for children with overweight and obesity: study protocol for a randomized controlled trial. *Trials.* 2020;21(1):132.

76. Moores CJ, Maeder A, Miller J, et al. A Digital Intervention for Australian Adolescents Above a Healthy Weight (Health Online for Teens): Protocol for an Implementation and User Experience Study. *JMIR Res Protoc.* 2019;8(10):e13340.

77. Moorhead A, Coates V, Hazlett D, et al. Weight Care Project: Health professionals' attitudes and ability to assess body weight status - study protocol. *BMC public health.* 2011;11:202.

78. Morgan PJ, Collins CE, Plotnikoff RC, et al. The SHED-IT community trial study protocol: a randomised controlled trial of weight loss programs for overweight and obese men. *BMC public health.* 2010;10:701.

79. O'Malley G, Clarke M, Burls A, Murphy S, Murphy N, Perry IJ. A smartphone intervention for adolescent obesity: study protocol for a randomised controlled non-inferiority trial. *Trials.* 2014;15:43.

80. Parkinson KN, Jones AR, Tovee MJ, et al. A cluster randomised trial testing an intervention to improve parents' recognition of their child's weight status: study protocol. *BMC Public Health.* 2015;15:549.

81. Parretti HM, Ives NJ, Tearne S, et al. Protocol for the feasibility and acceptability of a brief routine weight management intervention for postnatal women embedded within the national child immunisation programme: randomised controlled cluster feasibility trial with nested qualitative study (PIMMS-WL). *BMJ Open.* 2020;10(2):e033027.

82. Pbert L, Trivedi M, Druker S, et al. Supporting families of children with overweight and obesity to live healthy lifestyles: Design and rationale for the Fitline cluster randomized controlled pediatric practice-based trial. *Contemp Clin Trials.* 2021;104:106348.

83. Ramalho S, Saint-Maurice PF, Silva D, et al. APOLO-Teens, a web-based intervention for treatment-seeking adolescents with overweight or obesity: study protocol and baseline characterization of a Portuguese sample. *Eat Weight Disord.* 2020;25(2):453-463.

84. Shrewsbury VA, O'Connor J, Steinbeck KS, et al. A randomised controlled trial of a community-based healthy lifestyle program for overweight and obese adolescents: the Loozit study protocol. *BMC Public Health.* 2009;9:119.

85. Timpel P, Cesena FHY, Costa CD, et al. Efficacy of gamification-based smartphone application for weight loss in overweight and obese adolescents: study protocol for a phase II randomized controlled trial. *THERAPEUTIC ADVANCES IN ENDOCRINOLOGY AND METABOLISM.* 2018;9(6):167-176.

86. Tragomalou A, Kassari P, Ioakeimidis I, et al. BigO: The use of new technologies for the management of childhood obesity-A clinical pilot study. *Hormone Research in Paediatrics.* 2019;91:544.

87. Tragomalou A, Kassari P, Ioakeimidis I, et al. BigO: Novel technologies for the management of childhood obesity-The case of a clinical study in Greece. *Obesity Reviews.* 2020;21(SUPPL 1).

88. Wake M, Lycett K, Sabin MA, et al. A shared-care model of obesity treatment for 3-10 year old children: protocol for the HopSCOTCH randomised controlled trial. *BMC Pediatr.* 2012;12:39.

89. Willeboordse M, van de Kant KD, de Laat MN, van Schayck OC, Mulkens S, Dompeling E. Multifactorial intervention for children with asthma and overweight (Mikado): study design of a randomised controlled trial. *BMC Public Health.* 2013;13:494.

90. Wright ME, Delacroix E, Sonneville KR, et al. Reducing paediatric overweight and obesity through motivational interviewing: study protocol for a randomised controlled trial in the AAP PROS research network. *BMJ Open.* 2020;10(7):e035720.

91. Ansari MR, Kodriati N, Pertiwi AAP, Dewi FST. The Effectiveness of a Telenutrition Intervention to Improve Dietary Behavior and Physical Activity Among Adolescents With Obesity: Protocol for a Systematic Review. *JMIR Res Protoc.* 2024;13:e53282.

92. Assemany CG, Cunha DB, Brandão JM, et al. A multicomponent family intervention, combined with salt reduction for children with obesity: a factorial randomized study protocol. *BMC Public Health.* 2023;23(1):1453.

93. Barlow SE, Yudkin J, Nelson V, Allicock MA. Dynamo Kids!/¡Niños Dinámicos! A Web Site for Pediatric Primary Care Providers to Offer Parents of Children 6-12 Years Old With Overweight and Obesity: Web Site Development and Protocol for Pilot Study. *J Pediatr Health Care.* 2023;37(1):17-24.

94. Bernhardsson S, Boman C, Lundqvist S, et al. Implementation of physical activity on prescription for children with obesity in paediatric health care (IMPA): protocol for a feasibility and evaluation study using quantitative and qualitative methods. *Pilot Feasibility Stud.* 2022;8(1):117.

95. Cunningham PB, Naar S, Roberts JR, et al. Study protocol for clinical trial of the FIT Families multicomponent obesity intervention for African American adolescents and their caregivers: Next step from the ORBIT initiative. *BMJ Open.* 2024;14(2):e074552.

96. Davis AM, Befort CA, Lancaster BD, et al. Rationale and design of integrating a parents first obesity intervention with a pediatric weight management intervention for rural families - Evaluating the ripple effect. *Contemp Clin Trials.* 2023;128:107140.

97. Neshteruk CD, Skinner AC, Counts J, et al. Translating knowledge into action for child obesity treatment in partnership with Parks and Recreation: study protocol for a hybrid type II trial. *Implement Sci.* 2023;18(1):6.

98. O'Reilly SL, Burden C, Campoy C, et al. Bump2Baby and Me: protocol for a randomised trial of mHealth coaching for healthy gestational weight gain and improved postnatal outcomes in high-risk women and their children. *Trials.* 2021;22(1):963.

99. Pare SM, Gunn E, Morrison KM, et al. Testing a Biobehavioral Model of Chronic Stress and Weight Gain in Young Children (Family Stress Study): Protocol and Baseline Demographics for a Prospective Observational Study. *JMIR Res Protoc.* 2024;13:e48549.

100. Tian C, Xu J, Wang G, Yu L, Tang X. The effectiveness of web-based interventions on non-alcoholic fatty liver disease (NAFLD) in obese children: A study protocol for a randomized controlled trial. *Front Public Health.* 2022;10:930901.

101. Zare Z, Hajizadeh E, Mahmoodi M, Nazari R, Shahmoradi L, Rezayi S. Smartphone-based application to control and prevent overweight and obesity in children: design and evaluation. *BMC Med Inform Decis Mak.* 2023;23(1):201.

102. Zhu D, Dordevic AL, Gibson S, Davidson ZE. Evaluating a 10-Week Family-Focused E-Health Healthy Lifestyle Program for School-Aged Children with Overweight or Obesity: A Randomized Controlled Trial Study Protocol. *Nutrients.* 2023;15(13).

103. Mani S, Joseph LH, Sharma S. Feasibility of telemedicine or telephone-based family intervention for rural paediatric obesity: Cluster randomized control trial. *J Telemed Telecare*. Jun 2016;22(4):264-5. doi:10.1177/1357633x15601524

**Comparison between different tele-interventions (i = 9)**

104. Abraham AA, Chow WC, So HK, Woo J, Chan SM, Nelson EAS. Feasibility of Using Cell Phone Reminders to Motivate Behaviour Change in Obese Adolescents in Hong Kong. *Hong Kong Journal of Paediatrics.* 2014;19(3):197.

105. Chen JL, Guedes CM, Cooper BA, Lung AE. Short-Term Efficacy of an Innovative Mobile Phone Technology-Based Intervention for Weight Management for Overweight and Obese Adolescents: Pilot Study. *Interact J Med Res.* 2017;6(2):e12.

106. Chen JL, Guedes CM, Lung AE. Smartphone-based Healthy Weight Management Intervention for Chinese American Adolescents: Short-term Efficacy and Factors Associated With Decreased Weight. *J Adolesc Health.* 2019;64(4):443-449.

107. Davis AM, Sampilo M, Gallagher KS, et al. Treating rural paediatric obesity through telemedicine vs. telephone: Outcomes from a cluster randomized controlled trial. *J Telemed Telecare.* 2016;22(2):86-95.

108. Maddison R, Foley L, Ni Mhurchu C, et al. Effects of active video games on body composition: a randomized controlled trial. *Am J Clin Nutr.* 2011;94(1):156-163.

109. Taveras EM, Marshall R, Sharifi M, et al. Comparative Effectiveness of Clinical-Community Childhood Obesity Interventions: A Randomized Clinical Trial. *JAMA Pediatr.* 2017;171(8):e171325.

110. Tully L, Sorensen J, O'Malley G. Pediatric Weight Management Through mHealth Compared to Face-to-Face Care: Cost Analysis of a Randomized Control Trial. *JMIR Mhealth Uhealth.* 2021;9(9):e31621.

111. Wilson DK, Sweeney AM, Van Horn ML, et al. The Results of the Families Improving Together (FIT) for Weight Loss Randomized Trial in Overweight African American Adolescents. *Ann Behav Med.* 2022;56(10):1042-1055.

112. Zhang Q, O'Connor DB, Hugh-Jones S. Feasibility of a multiple-component mindfulness intervention for Chinese adolescents living with overweight: A pilot randomized trial. *Appl Psychol Health Well Being.* 2023;15(2):516-535.

**Systematic review (i = 65)**

113. Ajie WN, Chapman-Novakofski KM. Impact of computer-mediated, obesity-related nutrition education interventions for adolescents: a systematic review. *J Adolesc Health.* 2014;54(6):631-645.

114. Altman M, Wilfley DE. Evidence update on the treatment of overweight and obesity in children and adolescents. *J Clin Child Adolesc Psychol.* 2015;44(4):521-537.

115. An JY, Hayman LL, Park YS, Dusaj TK, Ayres CG. Web-based weight management programs for children and adolescents: a systematic review of randomized controlled trial studies. *ANS Adv Nurs Sci.* 2009;32(3):222-240.

116. Antwi F, Fazylova N, Garcon MC, Lopez L, Rubiano R, Slyer JT. The effectiveness of web-based programs on the reduction of childhood obesity in school-aged children: A systematic review. *JBI Libr Syst Rev.* 2012;10(42 Suppl):1-14.

117. Antwi FA, Fazylova N, Garcon MC, Lopez L, Rubiano R, Slyer JT. Effectiveness of web-based programs on the reduction of childhood obesity in school-aged children: A systematic review. *JBI Database of Systematic Reviews and Implementation Reports.* 2013;11(6):1-44.

118. Azevedo LB, Stephenson J, Ells L, et al. The effectiveness of e-health interventions for the treatment of overweight or obesity in children and adolescents: A systematic review and meta-analysis. *Obes Rev.* 2021:e13373.

119. Baker STE, Yucel M, Fornito A, Allen NB, Lubman DI. A systematic review of diffusion weighted MRI studies of white matter microstructure in adolescent substance users. *NEUROSCIENCE AND BIOBEHAVIORAL REVIEWS.* 2013;37(8):1713-1723.

120. Bernardi L, Novello D. Analysis of the influence of television on the levels of obesity in adolescents: a systematic review. *RBONE-REVISTA BRASILEIRA DE OBESIDADE NUTRICAO E EMAGRECIMENTO.* 2017;11(64):199-210.

121. Bryant MJ, Lucove JC, Evenson KR, Marshall S. Measurement of television viewing in children and adolescents: A systematic review. *Obesity Reviews.* 2007;8(3):197-209.

122. Chang TH, Chen YC, Chen WY, et al. Weight Gain Associated with COVID-19 Lockdown in Children and Adolescents: A Systematic Review and Meta-Analysis. *Nutrients.* 2021;13(10).

123. Chaplais E, Naughton G, Thivel D, Courteix D, Greene D. Smartphone Interventions for Weight Treatment and Behavioral Change in Pediatric Obesity: A Systematic Review. *Telemed J E Health.* 2015;21(10):822-830.

124. Cliff DP, Ridgers ND, Tsiros MD, et al. Associations between objectively measured sedentary behaviour and adiposity in children and adolescents: Systematic review and meta-analysis. *Journal of Science and Medicine in Sport.* 2014;18:e154.

125. DeSilva S, Vaidya SS. The Application of Telemedicine to Pediatric Obesity: Lessons from the Past Decade. *Telemed J E Health.* 2021;27(2):159-166.

126. Duriancik DM, Goff CR. Children of single-parent households are at a higher risk of obesity: A systematic review. *J Child Health Care.* 2019;23(3):358-369.

127. Faulkner GEJ, Buliung RN, Flora PK, Fusco C. Active school transport, physical activity levels and body weight of children and youth: A systematic review. *PREVENTIVE MEDICINE.* 2009;48(1):3-8.

128. Ferreira MS, Marson FAL, Wolf VLW, Ribeiro JD, Mendes RT. Lung function in obese children and adolescents without respiratory disease: a systematic review. *BMC Pulm Med.* 2020;20(1):281.

129. Ghobadi S, Hassanzadeh-Rostami Z, Salehi-Marzijarani M, et al. Association of eating while television viewing and overweight/obesity among children and adolescents: a systematic review and meta-analysis of observational studies. *Obes Rev.* 2018;19(3):313-320.

130. Hamel LM, Robbins LB, Wilbur J. Computer- and web-based interventions to increase preadolescent and adolescent physical activity: A systematic review. *Journal of Advanced Nursing.* 2011;67(2):251-268.

131. Hammersley ML, Jones RA, Okely AD. Parent-Focused Childhood and Adolescent Overweight and Obesity eHealth Interventions: A Systematic Review and Meta-Analysis. *J Med Internet Res.* 2016;18(7):e203.

132. Jesus GDS, Costa PRF, Oliveira LPM, et al. Body Adiposity and Apolipoproteins in Children and Adolescents: A Meta-Analysis of Prospective Studies. *Arq Bras Cardiol.* 2020;115(2):163-171.

133. Kaakinen P, Kyngäs H, Kääriäinen M. Technology-based counseling in the management of weight and lifestyles of obese or overweight children and adolescents: A descriptive systematic literature review. *Inform Health Soc Care.* 2018;43(2):126-141.

134. Kracht CL, Hutchesson M, Ahmed M, et al. E-&mHealth interventions targeting nutrition, physical activity, sedentary behavior, and/or obesity among children: A scoping review of systematic reviews and meta-analyses. *OBESITY REVIEWS.* 2021;22(12).

135. Lampe EW, Abber SR, Forman EM, Manasse SM. Guidelines for caregivers and healthcare professionals on speaking to children about overweight and obesity: a systematic review of the gray literature. *Transl Behav Med.* 2020;10(5):1144-1154.

136. Langarizadeh M, Sadeghi M, As'habi A, Rahmati P, Sheikhtaheri A. Mobile apps for weight management in children and adolescents; An updated systematic review. *Patient Educ Couns.* 2021;104(9):2181-2188.

137. Lee J, Piao M, Byun A, Kim J. A Systematic Review and Meta-Analysis of Intervention for Pediatric Obesity Using Mobile Technology. *Studies in health technology and informatics.* 2016;225:491-494.

138. Margetin CA, Rigassio Radler D, Thompson K, et al. Anthropometric Outcomes of Children and Adolescents Using Telehealth with Weight Management Interventions Compared to Usual Care: A Systematic Review and Meta-analysis. *J Am Coll Nutr.* 2021:1-24.

139. Marker C, Gnambs T, Appel M. Exploring the myth of the chubby gamer: A meta-analysis on sedentary video gaming and body mass. *Social Science and Medicine.* 2019.

140. Masoumi HE. ACTIVE TRANSPORT TO SCHOOL AND CHILDREN'S BODY WEIGHT A SYSTEMATIC REVIEW. *TEMA-JOURNAL OF LAND USE MOBILITY AND ENVIRONMENT.* 2017;10(1):95-110.

141. McGavock J, Chauhan BF, Rabbani R, et al. Layperson-Led vs Professional-Led Behavioral Interventions for Weight Loss in Pediatric Obesity: A Systematic Review and Meta-analysis. *JAMA Netw Open.* 2020;3(7):e2010364.

142. McMullan M, Millar R, Woodside JV. A systematic review to assess the effectiveness of technology-based interventions to address obesity in children. *BMC Pediatr.* 2020;20(1):242.

143. Meidani Z, Nabovati E, Gohari S, Chopannejad S. Phone-based interventions to control obesity in children under six years of age: A systematic review on features and effects. *Journal of Comprehensive Pediatrics.* 2018;9(3).

144. Mitchell TB, Amaro CM, Steele RG. Pediatric Weight Management Interventions in Primary Care Settings: A Meta-Analysis. *Health Psychol.* 2016.

145. Moorman EL, Koskela-Staples NC, Mathai BB, Fedele DA, Janicke DM. Pediatric Obesity Treatment via Telehealth: Current Evidence and Future Directions. *Curr Obes Rep.* 2021;10(3):371-384.

146. Neves MEA, de Souza MR, Gorgulho BM, Cunha DB, Muraro AP, Rodrigues PRM. Association of dietary patterns with blood pressure and body adiposity in adolescents: a systematic review. *EUROPEAN JOURNAL OF CLINICAL NUTRITION.* 2021;75(10):1440-1453.

147. O'Brien A. "Web-based weight management programs for children and adolescents: a systematic review of randomized controlled trial studies" by An, Hayman, Park, Dusaj, and Ayres (July-September 2009, Vol 32, No 3, pp 222-240). *ANS Adv Nurs Sci.* 2010;33(1):2.

148. Perego P, Rashid R, Gluud C, Jakobsen JC, Andreoni G, Lissau I. Comparison of different mobile health applications for intervention in children and adolescent with overweight: a protocol for systematic review with meta-analysis and trial sequential analysis. *BMJ Open.* 2020;10(12):e032570.

149. Pourmoradian S, Ostadrahimi A, Bonab AM, Roudsari AH, Jabbari M, Irandoost P. Television food advertisements and childhood obesity: A systematic review. *International Journal for Vitamin and Nutrition Research.* 2021;91(1-2):3-9.

150. Rashid R, Perego P, Condon L, et al. Health apps targeting children with overweight-a protocol for a systematic review with meta-analysis and Trial Sequential Analysis of randomised clinical trials. *Syst Rev.* 2020;9(1):28.

151. Reddy P, Dukhi N, Sewpaul R, Ellahebokus MAA, Kambaran NS, Jobe W. Mobile Health Interventions Addressing Childhood and Adolescent Obesity in Sub-Saharan Africa and Europe: Current Landscape and Potential for Future Research. *Front Public Health.* 2021;9:604439.

152. Rossi CE, Albernaz DO, de Vasconcelos FDG, de Assis MAA, Di Pietro PF. Television influence on food intake and obesity in children and adolescents: a systematic review. *REVISTA DE NUTRICAO-BRAZILIAN JOURNAL OF NUTRITION.* 2010;23(4):607-620.

153. Santos REA, Lacerda DC, da Silva MG, Barbosa DAM, Pinheiro IL, Pereira KNF. Mastication in children and adolescents with overweight or obesity: a systematic review. *REVISTA DE NUTRICAO-BRAZILIAN JOURNAL OF NUTRITION.* 2021;34.

154. Sonntag D, Schneider S, Mdege N, Ali S, Schmidt B. Beyond Food Promotion: A Systematic Review on the Influence of the Food Industry on Obesity-Related Dietary Behaviour among Children. *Nutrients.* 2015;7(10):8565-8576.

155. Te Velde SJ, Van Nassau F, Uijtdewilligen L, et al. Energy balance-related behaviours associated with overweight and obesity in preschool children: A systematic review of prospective studies. *Obesity Reviews.* 2012;13(SUPPL. 1):56-74.

156. Vescio A, Testa G, Montemagno M, Sapienza M, Pavone V. Secondary displacement risk after reduction and cast immobilization of displaced distal radius fractures in overweight and obese children: a systematic review and meta-analysis. *MINERVA ORTHOPEDICS.* 2021;72(1):71-76.

157. Whitley A, Yahia N. Efficacy of Clinic-Based Telehealth vs. Face-to-Face Interventions for Obesity Treatment in Children and Adolescents in the United States and Canada: A Systematic Review. *Child Obes.* 2021;17(5):299-310.

158. Wickham CA, Carbone ET. Who's calling for weight loss? A systematic review of mobile phone weight loss programs for adolescents. *Nutr Rev.* 2015;73(6):386-398.

159. Azevedo LB, Stephenson J, Ells L, et al. The effectiveness of e-health interventions for the treatment of overweight or obesity in children and adolescents: A systematic review and meta-analysis. *Obes Rev.* 2022;23(2):e13373.

160. Bleiweiss-Sande R, Skelton K, Zaltz D, Bacardí-Gascón M, Jiménez-Cruz A, Benjamin-Neelon SE. Interventions to prevent obesity in Latinx children birth to 6 years globally: a systematic review. *Public Health Nutr.* 2023;26(11):2498-2513.

161. Chai LK, Farletti R, Fathi L, Littlewood R. A Rapid Review of the Impact of Family-Based Digital Interventions for Obesity Prevention and Treatment on Obesity-Related Outcomes in Primary School-Aged Children. *Nutrients.* 2022;14(22).

162. Davison GM, Monocello LT, Lipsey K, Wilfley DE. Evidence Base Update on Behavioral Treatments for Overweight and Obesity in Children and Adolescents. *J Clin Child Adolesc Psychol.* 2023;52(5):589-603.

163. Hassapidou M, Duncanson K, Shrewsbury V, et al. EASO and EFAD Position Statement on Medical Nutrition Therapy for the Management of Overweight and Obesity in Children and Adolescents. *Obes Facts.* 2023;16(1):29-52.

164. Henes ST, Stotz SA, Riggs SE, Yang HM. eHealth, family-based interventions, and multilevel approaches to pediatric weight management: a scoping review. *Nutr Rev.* 2024.

165. Kirk S, Ogata B, Wichert E, Handu D, Rozga M. Treatment of Pediatric Overweight and Obesity: Position of the Academy of Nutrition and Dietetics Based on an Umbrella Review of Systematic Reviews. *J Acad Nutr Diet.* 2022;122(4):848-861.

166. Kouvari M, Karipidou M, Tsiampalis T, et al. Digital Health Interventions for Weight Management in Children and Adolescents: Systematic Review and Meta-analysis. *J Med Internet Res.* 2022;24(2):e30675.

167. Lin C, Li D, Wang X, Yang S. Chronic exercise interventions for executive function in overweight children: a systematic review and meta-analysis. *Front Sports Act Living.* 2024;6:1336648.

168. Margetin CA, Rigassio Radler D, Thompson K, et al. Anthropometric Outcomes of Children and Adolescents Using Telehealth with Weight Management Interventions Compared to Usual Care: A Systematic Review and Meta-analysis. *J Am Nutr Assoc.* 2022;41(2):207-229.

169. Metzendorf MI, Wieland LS, Richter B. Mobile health (m-health) smartphone interventions for adolescents and adults with overweight or obesity. *Cochrane Database Syst Rev.* 2024;2(2):Cd013591.

170. Mirza M, Brown-Hollie JP, Suarez-Balcazar Y, et al. Interventions for Health Promotion and Obesity Prevention for Children and Adolescents with Developmental Disabilities: a Systematic Review. *Rev J Autism Dev Disord.* 2022:1-24.

171. O'Hara VM, Louder D, Johnston SV, Hastey K, Browne NT. Pediatric Obesity Care via Telemedicine: Expanding the Path Forward-A Review. *Curr Obes Rep.* 2023;12(4):546-556.

172. Pawellek S, Ziegeldorf A, Wulff H. [Strategies and effects of digital interventions in overweight and obesity treatments in children and adolescents-a systematic review]. *Bundesgesundheitsblatt Gesundheitsforschung Gesundheitsschutz.* 2022;65(5):624-634.

173. Soltero EG, Lopez C, Hernandez E, O'Connor TM, Thompson D. Technology-Based Obesity Prevention Interventions Among Hispanic Adolescents in the United States: Scoping Review. *JMIR Pediatr Parent.* 2022;5(4):e39261.

174. Vandoni M, Codella R, Pippi R, et al. Combatting Sedentary Behaviors by Delivering Remote Physical Exercise in Children and Adolescents with Obesity in the COVID-19 Era: A Narrative Review. *Nutrients.* 2021;13(12).

175. Yau KW, Tang TS, Görges M, et al. Effectiveness of Mobile Apps in Promoting Healthy Behavior Changes and Preventing Obesity in Children: Systematic Review. *JMIR Pediatr Parent.* 2022;5(1):e34967.

176. Zhang P, Tang X, Peng X, Hao G, Luo S, Liang X. Effect of screen time intervention on obesity among children and adolescent: A meta-analysis of randomized controlled studies. *Prev Med.* 2022;157:107014.

177. Zuñiga Vinueza AM, Jaramillo AP. The Effectiveness of a Healthy Lifestyle in Obese Pediatric Patients: A Systematic Review and Meta-Analysis. *Cureus.* 2023;15(11):e48525.

**Supplementary Table 4** Risk of bias (Cochrane risk-of-bias tool 2, RoB 2)

| **Outcome / Study** | **Domain 1** | **Domain 2** | **Domain 3** | **Domain 4** | **Domain 5** | **Overall** |
| --- | --- | --- | --- | --- | --- | --- |
| **Body mass index *z* score** | | | | | | |
| Ahmad et al. 2018 | Low risk | Low risk | Low risk | Low risk | Low risk | Low risk |
| Bovi et al. 2021 | Some concern | Low risk | Low risk | Low risk | Low risk | Some concern |
| Currie et al. 2018 | Low risk | Low risk | Low risk | Low risk | Low risk | Low risk |
| Estabrooks et al. 2009 | Low risk | Low risk | Some concern | Low risk | Low risk | Some concern |
| Fleischman et al. 2016 | Low risk | Low risk | Low risk | Low risk | Low risk | Low risk |
| Johansson et al. 2020 | Low risk | Low risk | High risk | Low risk | Low risk | High risk |
| Love Osborn et al. 2014 | Low risk | Low risk | Low risk | Low risk | Low risk | Low risk |
| Nguyen et al. 2013 | Some concern | Some concern | High risk | Low risk | Low risk | High risk |
| Niet et al. 2012 | Low risk | Low risk | Low risk | Low risk | Low risk | Low risk |
| Patrick et al. 2013 | Low risk | Low risk | Low risk | Low risk | Low risk | Low risk |
| Staiano et al. 2018 | Low risk | Low risk | Low risk | Low risk | Low risk | Low risk |
| Stasinaki et al. 2021 | Low risk | Low risk | Low risk | Low risk | Low risk | Low risk |
| Taveras et al. 2015 | Some concern | Low risk | Low risk | Low risk | Low risk | Some concern |
| Vermeiren et al. 2021 | Low risk | Low risk | Low risk | Low risk | Low risk | Low risk |
| Vidmar et al. 2021 | Low risk | Low risk | Low risk | Low risk | Low risk | Low risk |
| Wake et al. 2013 | Low risk | Low risk | Low risk | Low risk | Low risk | Low risk |
| Wald et al. 2017 | Some concern | Low risk | High risk | Low risk | Low risk | High risk |
| **Body mass index** | | | | | | |
| Abraham et al. 2015 | Low risk | Low risk | Low risk | Low risk | Some concern | Some concern |
| Bovi et al. 2021 | Some concern | Low risk | Low risk | Low risk | Low risk | Some concern |
| Fleischman et al. 2016 | Low risk | Low risk | Low risk | Low risk | Low risk | Low risk |
| Lee et al. 2017 | Low risk | Low risk | Low risk | Low risk | Low risk | Low risk |
| Likhitweerawong et al. 2020 | Low risk | Low risk | Low risk | Low risk | Low risk | Low risk |
| Nawi et al. 2015 | Some concern | Low risk | Low risk | Low risk | Low risk | Some concern |
| Nguyen et al. 2013 | Some concern | Some concern | High risk | Low risk | Low risk | High risk |
| Ptomey et al. 2021 | Low risk | Low risk | Low risk | Low risk | Low risk | Low risk |
| Taveras et al. 2015 | Some concern | Low risk | Low risk | Low risk | Low risk | Some concern |
| Tsiros et al. 2008 | Low risk | Low risk | Low risk | Low risk | Low risk | Low risk |
| Wald et al. 2017 | Some concern | Low risk | High risk | Low risk | Low risk | High risk |

Domain 1: bias raising from the randomization process

Domain 2: bias due to deviation from intended interventions

Domain 3: bias due to missing outcome data

Domain 4: bias in measurement of outcome

Domain 5: bias in selection of the reported result

**Supplementary Table 4 (continued)** Risk of bias (Cochrane risk-of-bias tool 2, RoB 2)

| **Outcome / Study** | **Domain 1** | **Domain 2** | **Domain 3** | **Domain 4** | **Domain 5** | **Overall** |
| --- | --- | --- | --- | --- | --- | --- |
| **Body fat** | | | | | | |
| Abraham et al. 2015 | Low risk | Low risk | Low risk | Low risk | Some concern | Some concern |
| Ahmad et al. 2018 | Low risk | Low risk | Low risk | Low risk | Low risk | Low risk |
| Lee et al. 2017 | Low risk | Low risk | Low risk | Low risk | Low risk | Low risk |
| Nawi et al. 2015 | Some concern | Low risk | Low risk | Low risk | Low risk | Some concern |
| Patrick et al. 2013 | Low risk | Low risk | Low risk | Low risk | Low risk | Low risk |
| Staiano et al. 2018 | Low risk | Low risk | Low risk | Low risk | Low risk | Low risk |
| Stasinaki et al. 2021 | Low risk | Low risk | Low risk | Low risk | Low risk | Low risk |
| Tsiros et al. 2008 | Low risk | Low risk | Low risk | Low risk | Low risk | Low risk |
| Wake et al. 2013 | Low risk | Low risk | Low risk | Low risk | Low risk | Low risk |
| **Waist circumference** | | | | | | |
| Abraham et al. 2015 | Low risk | Low risk | Low risk | Low risk | Some concern | Some concern |
| Ahmad et al. 2018 | Low risk | Low risk | Low risk | Low risk | Low risk | Low risk |
| Bovi et al. 2021 | Some concern | Low risk | Low risk | Low risk | Low risk | Some concern |
| Fleischman et al. 2016 | Low risk | Low risk | Low risk | Low risk | Low risk | Low risk |
| Likhitweerawong et al. 2020 | Low risk | Low risk | Low risk | Low risk | Low risk | Low risk |
| Nguyen et al. 2013 | Some concern | Some concern | High risk | Low risk | Low risk | High risk |
| Ptomey et al. 2021 | Low risk | Low risk | Low risk | Low risk | Low risk | Low risk |
| Stasinaki et al. 2021 | Low risk | Low risk | Low risk | Low risk | Low risk | Low risk |
| Tsiros et al. 2008 | Low risk | Low risk | Low risk | Low risk | Low risk | Low risk |
| Wake et al. 2013 | Low risk | Low risk | Low risk | Low risk | Low risk | Low risk |

Domain 1: bias raising from the randomization process

Domain 2: bias due to deviation from intended interventions

Domain 3: bias due to missing outcome data

Domain 4: bias in measurement of outcome

Domain 5: bias in selection of the reported result

**Supplementary Table 5** Summary of studies for each outcome

| **Measure** | **Time Frame** | **Number of RCTs** | **Study References** |
| --- | --- | --- | --- |
| BMI z-score | Within 3 months | 7 | 8, 9, 11, 13, 14, 26, 27 |
|  | Within 6 months | 10 | 6, 8, 11-14, 20, 22, 23, 26 |
|  | Over 6 months | 12 | 6, 7, 11, 12, 17, 19, 20, 23, 24, 26, 28, 29 |
| BMI | Within 3 months | 6 | 5, 8, 13, 16, 18, 25 |
|  | Within 6 months | 6 | 5, 8, 13, 15, 21, 25 |
|  | Over 6 months | 4 | 19, 24, 28, 29 |
| Body fat | Within 3 months | 2 | 5, 18 |
|  | Within 6 months | 7 | 5, 6, 15, 20, 22, 23, 25 |
|  | Over 6 months | 4 | 6, 20, 23, 28 |
| Waist circumference | Within 3 months | 5 | 5, 8, 13, 16, 18 |
|  | Within 6 months | 7 | 5, 6, 8, 13, 21, 23, 25 |
|  | Over 6 months | 4 | 5, 19, 22, 28 |

RCT, randomized controlled trial. Note the reference numbers are based on the reference in the main text.

**Supplementary Table 5** Summary of finding and certainty evaluation

| **No. of participants** | **Certainty of the** | **Relative effects** |  |
| --- | --- | --- | --- |
| **(studies)** | **Evidence (GRADE)** | **(95% CI)** | **Comments** |
| **Body mass index *z*-score within 3 months** | | | |
| 561  (7 RCTs) | ⨁⨁⨁◯ ^a^  MODERATE | **MD -0.09**  (-0.20 to 0.01) | Tele-interventions do not decrease BMI z-score in three months. |
| **Body mass index within 3 months** | | | |
| 405  (6 RCTs) | ⨁⨁◯◯ ^a,b^  LOW | **MD -0.64**  (-1.24 to -0.04) | Tele-interventions slightly decrease BMI in three months. |
| **Body fat within 3 months** | | | |
| 145  (2 RCTs) | ⨁⨁◯◯ ^b,c^  LOW | **SMD -0.15**  (-0.60 to 0.30) | Tele-interventions do not decrease body fat in three months. |
| **Waist circumference within 3 months** | | | |
| 358  (5 RCTs) | ⨁◯◯◯ ^a,b,c,d^  VERY LOW | **SMD -0.40**  (-0.79 to -0.01) | Tele-interventions decrease waist circumference in three months. |
| **Body mass index *z*-score within 6 months** | | | |
| 876  (10 RCTs) | ⨁⨁⨁◯ ^a^  MODERATE | **MD -0.15**  (-0.23 to -0.08) | Tele-interventions slightly decrease BMI z-score in six months. |
| **Body mass index within 6 months** | | | |
| 457  (6 RCTs) | ⨁⨁◯◯ ^a,b,c,e^  LOW | **MD -2.48**  (-4.15 to -0.82) | Tele-interventions decrease BMI in six months. |
| **Body fat within 6 months** | | | |
| 489  (7 RCTs) | ⨁⨁⨁◯ ^a^  MODERATE | **SMD -0.26**  (-0.62 to 0.10) | Tele-interventions do not decrease body fat in six months. |
| **Waist circumference within 6 months** | | | |
| 478  (7 RCTs) | ⨁⨁⨁◯ ^a,e^  MODERATE | **SMD -0.59**  (-1.05 to -0.14) | Tele-interventions decrease waist circumference in six months. |
| **Body mass index *z*-score over 6 months** | | | |
| 1734  (12 RCTs) | ⨁◯◯◯ ^a,b,d^  VERY LOW | **MD -0.19**  (-0.34 to -0.03) | Tele-interventions slightly decrease BMI z-score after six months. |
| **Body mass index over 6 months** | | | |
| 816  (4 RCTs) | ⨁⨁◯◯ ^b,c^  LOW | **MD -0.06**  (-0.61 to 0.48) | Tele-interventions slightly decrease BMI after six months. |
| **Body fat over 6 months** | | | |
| 378  (4 RCTs) | ⨁⨁◯◯ ^b,c^  LOW | **SMD -0.22**  (-0.79 to 0.34) | Tele-interventions do not decrease body fat after six months. |
| **Waist circumference over 6 months** | | | |
| 387  (4 RCTs) | ⨁◯◯◯ ^a,b,c,d^  VERY LOW | **SMD -0.01**  (-0.40 to 0.39) | Tele-interventions do not decrease waist circumference in after six months. |

CI, confidence interval; MD, mean difference; SMD, standardized mean difference.

^a,^ Downgrade a level due to some concerns on heterogeneity (I-square > 50%).

^b,^ Downgrade a level due to contribution from some concerns (> 50%) or high risk of bias (> 25%) trials.

^c,^ Downgrade a level due to wide range of confidence interval and relatively small sample size.

^d,^ Downgrade two levels due to major concerns on heterogeneity with large contrary effects among trials.

^e,^ Upgrade a level due to relatively large effect size.

**Supplementary Figure 1** Forest plot of body mass index at baseline

**Supplementary Figure 2** Forest plot of body fat at baseline

**Supplementary Figure 3** Forest plot of waist circumference at baseline

**Supplementary Figure 4** Forest plot of BMI z-score at baseline

**Supplementary Figure 5** Forest plot of adherence rate


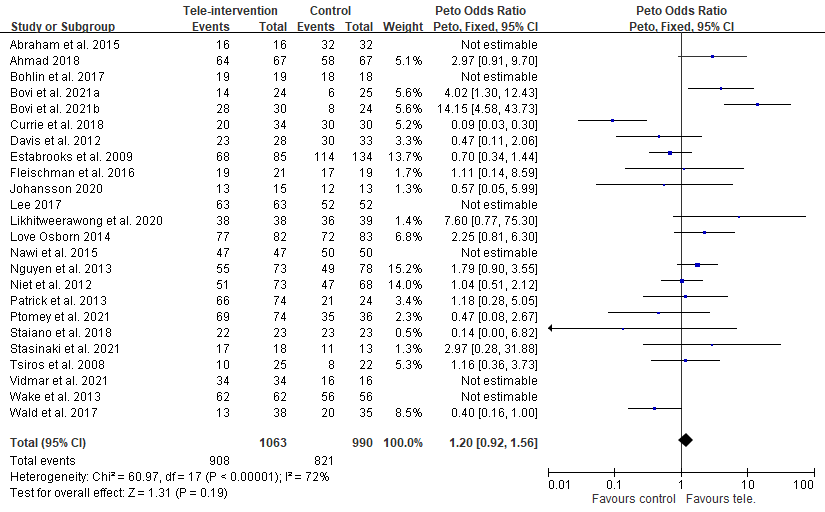


**Reference for the supplementary files**

1. Armstrong B, Trude ACB, Johnson C, et al. CHAMP: A cluster randomized-control trial to prevent obesity in child care centers. *Contemp Clin Trials*. Nov 2019;86:105849. doi:10.1016/j.cct.2019.105849

2. Arthurs N, Browne S, Boardman R, et al. The BigO application: Usability and engagement among adolescents and children with obesity. Conference Abstract. *Obesity Reviews*. 2020;21(SUPPL 1)doi:10.1111/obr.13118

3. Askie LM, Espinoza D, Martin A, et al. Interventions commenced by early infancy to prevent childhood obesity—The EPOCH Collaboration: An individual participant data prospective meta-analysis of four randomized controlled trials. Article. *Pediatric Obesity*. 2020;15(6)doi:10.1111/ijpo.12618

4. Austin SB, Spadano-Gasbarro JL, Greaney ML, et al. Effect of the planet health intervention on eating disorder symptoms in Massachusetts middle schools, 2005-2008. Article. *Preventing chronic disease*. 2012;9:E171; quiz E171. doi:10.5888/pcd9.120111

5. Brambilla P, Bedogni G, Buongiovanni C, et al. "Mi voglio bene": a pediatrician-based randomized controlled trial for the prevention of obesity in Italian preschool children. *Ital J Pediatr*. Aug 17 2010;36:55. doi:10.1186/1824-7288-36-55

6. Byrd-Bredbenner C, Martin-Biggers J, Koenings M, Quick V, Hongu N, Worobey J. HomeStyles, A Web-Based Childhood Obesity Prevention Program for Families With Preschool Children: Protocol for a Randomized Controlled Trial. *JMIR Res Protoc*. Apr 25 2017;6(4):e73. doi:10.2196/resprot.7544

7. Byrd-Bredbenner C, Santiago E, Eck KM, et al. HomeStyles-2: Randomized controlled trial protocol for a web-based obesity prevention program for families with children in middle childhood. *Contemp Clin Trials*. Nov 30 2021:106644. doi:10.1016/j.cct.2021.106644

8. Cruz TH, Davis SM, FitzGerald CA, Canaca GF, Keane PC. Engagement, recruitment, and retention in a trans-community, randomized controlled trial for the prevention of obesity in rural American Indian and Hispanic children. *J Prim Prev*. Jun 2014;35(3):135-49. doi:10.1007/s10935-014-0340-9

9. French SA, Gerlach AF, Mitchell NR, Hannan PJ, Welsh EM. Household obesity prevention: Take actiona group-randomized trial. Article. *Obesity*. 2011;19(10):2082-2088. doi:10.1038/oby.2010.328

10. Haines J, McDonald J, O'Brien A, et al. Healthy Habits, Happy Homes: randomized trial to improve household routines for obesity prevention among preschool-aged children. *JAMA Pediatr*. Nov 2013;167(11):1072-9. doi:10.1001/jamapediatrics.2013.2356

11. Hammersley ML, Jones RA, Okely AD. Time2bHealthy - An online childhood obesity prevention program for preschool-aged children: A randomised controlled trial protocol. *Contemp Clin Trials*. Oct 2017;61:73-80. doi:10.1016/j.cct.2017.07.022

12. Hammersley ML, Okely AD, Batterham MJ, Jones RA. An Internet-Based Childhood Obesity Prevention Program (Time2bHealthy) for Parents of Preschool-Aged Children: Randomized Controlled Trial. *J Med Internet Res*. Feb 8 2019;21(2):e11964. doi:10.2196/11964

13. Horodynski MA, Olson B, Baker S, et al. Healthy babies through infant-centered feeding protocol: an intervention targeting early childhood obesity in vulnerable populations. Article. *BMC public health*. 2011;11:868.

14. Knowlden A, Sharma M. One-Year Efficacy Testing of Enabling Mothers to Prevent Pediatric Obesity Through Web-Based Education and Reciprocal Determinism (EMPOWER) Randomized Control Trial. *Health Educ Behav*. Feb 2016;43(1):94-106. doi:10.1177/1090198115596737

15. Knowlden AP, Conrad E. Two-Year Outcomes of the Enabling Mothers to Prevent Pediatric Obesity Through Web-Based Education and Reciprocal Determinism (EMPOWER) Randomized Control Trial. *Health Educ Behav*. Apr 2018;45(2):262-276. doi:10.1177/1090198117732604

16. Knowlden AP, Sharma M. Process evaluation of the Enabling Mothers toPrevent Pediatric Obesity Through Web-Based Learning and Reciprocal Determinism (EMPOWER) randomized control trial. *Health Promot Pract*. Sep 2014;15(5):685-94. doi:10.1177/1524839914523431

17. Knowlden AP, Sharma M, Cottrell RR, Wilson BR, Johnson ML. Impact evaluation of Enabling Mothers to Prevent Pediatric Obesity through Web-Based Education and Reciprocal Determinism (EMPOWER) Randomized Control Trial. *Health Educ Behav*. Apr 2015;42(2):171-84. doi:10.1177/1090198114547816

18. Liu Z, Gao P, Gao AY, et al. Effectiveness of a Multifaceted Intervention for Prevention of Obesity in Primary School Children in China: A Cluster Randomized Clinical Trial. *JAMA Pediatr*. Nov 8 2021:e214375. doi:10.1001/jamapediatrics.2021.4375

19. Liu Z, Wu Y, Niu WY, et al. A school-based, multi-faceted health promotion programme to prevent obesity among children: protocol of a cluster-randomised controlled trial (the DECIDE-Children study). *BMJ Open*. Nov 2 2019;9(11):e027902. doi:10.1136/bmjopen-2018-027902

20. Lombard C, Deeks A, Jolley D, Ball K, Teede H. A low intensity, community based lifestyle programme to prevent weight gain in women with young children: cluster randomised controlled trial. *Bmj*. Jul 13 2010;341:c3215. doi:10.1136/bmj.c3215

21. Lubans DR, Smith JJ, Plotnikoff RC, et al. Assessing the sustained impact of a school-based obesity prevention program for adolescent boys: the ATLAS cluster randomized controlled trial. *Int J Behav Nutr Phys Act*. Aug 20 2016;13:92. doi:10.1186/s12966-016-0420-8

22. Markert J, Herget S, Petroff D, et al. Telephone-based adiposity prevention for families with overweight children (T.A.F.F.-Study): one year outcome of a randomized, controlled trial. *Int J Environ Res Public Health*. Oct 3 2014;11(10):10327-44. doi:10.3390/ijerph111010327

23. Nyström CD, Sandin S, Henriksson P, et al. Mobile-based intervention intended to stop obesity in preschool-aged children: the MINISTOP randomized controlled trial. *Am J Clin Nutr*. Jun 2017;105(6):1327-1335. doi:10.3945/ajcn.116.150995

24. Raat H, Struijk MK, Remmers T, et al. Primary prevention of overweight in preschool children, the BeeBOFT study (breastfeeding, breakfast daily, outside playing, few sweet drinks, less TV viewing): design of a cluster randomized controlled trial. *BMC public health*. Oct 19 2013;13doi:10.1186/1471-2458-13-974

25. Robinson TN. Reducing children's television viewing to prevent obesity - A randomized controlled trial. *JAMA-journal of the american medical association*. Oct 27 1999;282(16):1561-1567. doi:10.1001/jama.282.16.1561

26. Ruiter ELM, Fransen GAJ, Molleman GRM, van der Velden K, Engels R. The effectiveness of a web-based Dutch parenting program to prevent overweight in children 9-13 years of age: study protocol for a two-armed cluster randomized controlled trial. *BMC public health*. Feb 14 2015;15doi:10.1186/s12889-015-1394-1

27. Sherwood NE, Levy RL, Langer SL, et al. Healthy Homes/Healthy Kids: a randomized trial of a pediatric primary care-based obesity prevention intervention for at-risk 5-10 year olds. *Contemp Clin Trials*. Sep 2013;36(1):228-43. doi:10.1016/j.cct.2013.06.017

28. Simon C, Schweitzer B, Oujaa M, et al. Successful overweight prevention in adolescents by increasing physical activity: a 4-year randomized controlled intervention. *International journal of obesity*. Oct 2008;32(10):1489-1498. doi:10.1038/ijo.2008.99

29. Taveras EM, Gortmaker SL, Hohman KH, et al. Randomized controlled trial to improve primary care to prevent and manage childhood obesity: the High Five for Kids study. *Arch Pediatr Adolesc Med*. Aug 2011;165(8):714-22. doi:10.1001/archpediatrics.2011.44

30. van Grieken A, Vlasblom E, Wang L, et al. Personalized Web-Based Advice in Combination With Well-Child Visits to Prevent Overweight in Young Children: Cluster Randomized Controlled Trial. *J Med Internet Res*. Jul 27 2017;19(7):e268. doi:10.2196/jmir.7115

31. Veldhuis L, Struijk MK, Kroeze W, et al. 'Be active, eat right', evaluation of an overweight prevention protocol among 5-year-old children: design of a cluster randomised controlled trial. *BMC public health*. Jun 8 2009;9doi:10.1186/1471-2458-9-177

32. Verbestel V, De Coen V, Van Winckel M, Huybrechts I, Maes L, De Bourdeaudhuij I. Prevention of overweight in children younger than 2 years old: a pilot cluster-randomized controlled trial. *PUBLIC HEALTH NUTRITION*. JUN 2014;17(6):1384-1392. doi:10.1017/S1368980013001353

33. Weintraub DL, Tirumalai EC, Haydel KF, Fujimoto M, Fulton JE, Robinson TN. Team sports for overweight children - The Stanford sports to prevent obesity randomized trial (SPORT). *Archives of pediatrics & adolescent medicine*. Mar 2008;162(3):232-237. doi:10.1001/archpediatrics.2007.43

34. Wen LM, Rissel C, Baur LA, et al. A 3-Arm randomised controlled trial of Communicating Healthy Beginnings Advice by Telephone (CHAT) to mothers with infants to prevent childhood obesity. Article. *BMC public health*. 2017;17(1):79. doi:10.1186/s12889-016-4005-x

35. Wen LM, Rissel C, Xu HL, et al. Linking two randomised controlled trials for Healthy Beginnings (c): optimising early obesity prevention programs for children under 3years. *BMC public health*. Jun 13 2019;19doi:10.1186/s12889-019-7058-9

36. Wilken LR, Novotny R, Fialkowski MK, et al. Children's Healthy Living (CHL) Program for remote underserved minority populations in the Pacific region: rationale and design of a community randomized trial to prevent early childhood obesity. *BMC public health*. Oct 9 2013;13doi:10.1186/1471-2458-13-944

37. Adams b. Dose-response effects of an online and in-person family intervention on physical activity in children who are overweight. *Annals of behavioral medicine*. May 2020;54:S26-S26.

38. Alff F, Markert J, Zschaler S, Gausche R, Kiess W, Blüher S. Reasons for (non)participating in a telephone-based intervention program for families with overweight children. *PLoS One*. 2012;7(4):e34580. doi:10.1371/journal.pone.0034580

39. Bean MK, Mendoza A, Farthing S, Smith D, Adams EL, Caccavale L. Transitioning an adolescent obesity treatment from in-person to remote delivery: lessons learned from the teens plus trial. *Annals of behavioral medicine*. Apr 2021;55:S543-S543.

40. Bean MK, Thornton LM, Jeffers AJ, Gow RW, Mazzeo SE. Impact of motivational interviewing on engagement in a parent-exclusive paediatric obesity intervention: randomized controlled trial of NOURISH plus MI. *Pediatric obesity*. Apr 2019;14(4)doi:10.1111/ijpo.12484

41. Bovi APD, Cesari GM, Rocco MC, et al. Healthy lifestyle management of pediatric obesity with a hybrid system of customized mobile technology: The pediafit pilot project. Article. *Nutrients*. 2021;13(2):1-15. doi:10.3390/nu13020631

42. Browne S, Doyle G, Kechadi T, et al. Mobile health (mHealth) applications with children in treatment for obesity: A randomised feasibility study. Conference Abstract. *Proceedings of the Nutrition Society*. 2020;79(OCE2)doi:10.1017/S0029665120003420

43. Chew CSE, Davis C, Lim JKE, et al. Use of a Mobile Lifestyle Intervention App as an Early Intervention for Adolescents With Obesity: Single-Cohort Study. *J Med Internet Res*. Sep 28 2021;23(9):e20520. doi:10.2196/20520

44. Davis AM, James RL, Boles RE, Goetz JR, Belmont J, Malone B. The use of TeleMedicine in the treatment of paediatric obesity: feasibility and acceptability. *Matern Child Nutr*. Jan 2011;7(1):71-9. doi:10.1111/j.1740-8709.2010.00248.x

45. Davis AM, Sampilo M, Gallagher KS, et al. Treating rural paediatric obesity through telemedicine vs. telephone: Outcomes from a cluster randomized controlled trial. *Journal of telemedicine and telecare*. Mar 2016;22(2):86-95. doi:10.1177/1357633X15586642

46. Davis AM, Sampilo M, Gallagher KS, Landrum Y, Malone B. Treating rural pediatric obesity through telemedicine: outcomes from a small randomized controlled trial. *J Pediatr Psychol*. Oct 2013;38(9):932-43. doi:10.1093/jpepsy/jst005

47. DuBose KD, Currie J, Collier D, Raedeke TD, Kemble CD. The Effect of a Telephone-Based Physical Activity Intervention in Obese Adolescents. *Medicine and science in sports and exercise*. May 2014;46(5):170-170. doi:10.1249/01.mss.0000493688.62146.8d

48. Felix S, Ramalho S, Conceicao E, Saint-Maurice PF, Silva D, Mansilha HF. Satisfaction with Apolo-Teens Online Program for Overweight/Obese Adolescents. *Annals of nutrition and metabolism*. 2018;73(4):318-319.

49. Félix S, Ramalho S, Ribeiro E, Conceição E. Mothers and adolescents' experience regarding an online intervention for adolescents with overweight and obesity: A pilot study. Conference Abstract. *Obesity Reviews*. 2020;21(SUPPL 1)doi:10.1111/obr.13118

50. Felix S, Ramalho S, Ribeiro E, et al. Experiences of parent-adolescent dyads regarding a Facebook-based intervention to improve overweight/obesity treatment in adolescents: A qualitative study. *Applied psychology-health and well being*. doi:10.1111/aphw.12294

51. Jones A, Mann K, Cutler L, et al. Targeting parental recognition and understanding of childhood overweight to improve child weight outcomes: The impact of the MapMe intervention at 12 months follow-up. Conference Abstract. *Obesity Facts*. 2017;10:11-12. doi:10.1159/000468958

52. Kornman KP, Shrewsbury VA, Chou AC, et al. Electronic therapeutic contact for adolescent weight management: the Loozit study. *Telemed J E Health*. Jul-Aug 2010;16(6):678-85. doi:10.1089/tmj.2009.0180

53. L'Allemand D, Shih CH, Heldt K, et al. Design and interim evaluation of a smartphone app for overweight adolescents using a behavioural health intervention platform. Conference Abstract. *Obesity Reviews*. 2018;19:102. doi:10.1111/(ISSN)1467-789X

54. Lang L. Text Messaging May Help Children to Fight Off Obesity. Article. *Gastroenterology*. 2009;136(1):7-8. doi:10.1053/j.gastro.2008.11.047

55. Markert J, Herget S, Vogel M, Gausche R, Hilbert A, Blüher S. After care treatment approach for adolescent obesity via telephone counseling following an obesity treatment program-study concept. Conference Abstract. *Hormone Research in Paediatrics*. 2012;78:279. doi:10.1159/000343184

56. Nguyen B, Shrewsbury V, Lau C, et al. Adolescent and parent views of an adolescent weight management program: Lessons from the Loozit® randomised controlled trial. Conference Abstract. *Obesity Research and Clinical Practice*. 2012;6:56. doi:10.1016/j.orcp.2012.08.114

57. Nguyen B, Shrewsbury V, O'Connor J, et al. Two-year outcomes of an extended adolescent weight-loss maintenance intervention involving novel additional therapeutic contact: The Loozit® randomised controlled trial. Conference Abstract. *Obesity Research and Clinical Practice*. 2012;6:38-39. doi:10.1016/j.orcp.2012.08.079

58. Nguyen B, Shrewsbury V, O'Connor J, et al. Two-year outcomes of an extended adolescent weight-loss maintenance intervention involving novel additional therapeutic contact: The Loozit®R randomised controlled trial. Conference Abstract. *Obesity Facts*. 2012;5:188-189. doi:10.1159/000258190

59. Parra-Medina D, Mojica CM, Parma DL, Rubalcava L, Ramos AI. Preliminary results from an obesity management intervention in a rural pediatric practice: The NEST study. Conference Abstract. *Obesity*. 2011;19:S110. doi:10.1038/oby.2011.226

60. Ramalho S, Silva D, Mansilha H, Saint Maurice P, Conceição E. APOLO-Teens, a Web-based intervention for adolescents with overweight/obesity seeking treatment: An effectiveness study. Conference Abstract. *Obesity Facts*. 2019;12:74. doi:10.1159/000489691

61. Tabak RG, Tate DF, Stevens J, Siega-Riz AM, Ward DS. Family ties to health study: A randomized intervention to improve vegetable intake in children. Conference Abstract. *Obesity*. 2011;19:S109. doi:10.1038/oby.2011.226

62. Werk LN, Hossain J, Martinez A, et al. Extending obesity care beyond the office doors using telemedicine health coaches. A pilot test randomized trial. Conference Abstract. *Pediatrics*. 2019;144(2)doi:10.1542/peds.144.2-MeetingAbstract.219

63. Wu TJ, Holt NM, Dalton W, Maphis L, Schetzina K. A child weight management program targeting parents in primary care: insights from plan for healthy living phone follow-ups. *Annals of behavioral medicine*. Apr 2012;43:s194-s194.

64. Wyse R, Wolfenden L, Campbell E, et al. Increasing fruit and vegetable consumption in 3- 5 year old children: Results from a cluster randomised controlled trial of a telephone-based parent intervention, hunter region, NSW, Australia. Conference Abstract. *Obesity Reviews*. 2011;12:68. doi:10.1111/j.1467-789X.2011.00889.x

65. Beeken RJ, Croker H, Morris S, et al. Study protocol for the 10 Top Tips (10TT) trial: randomised controlled trial of habit-based advice for weight control in general practice. *BMC public health.* 2012;12:667.

66. Brennan L, Walkley J, Fraser SF, Greenway K, Wilks R. Motivational interviewing and cognitive behaviour therapy in the treatment of adolescent overweight and obesity: study design and methodology. *Contemp Clin Trials.* 2008;29(3):359-375.

67. De Assis MAA, Rolland-Cachera MF, De Vasconcelos FDAG, et al. Overweight and thinness in 7-9 year old children from Florianópolis, Southern Brazil: A comparison with a French study using a similar protocol. *Revista de Nutricao.* 2006;19(3):299-308.

68. Deehan EC, Colin-Ramirez E, Triador L, et al. Efficacy of metformin and fermentable fiber combination therapy in adolescents with severe obesity and insulin resistance: study protocol for a double-blind randomized controlled trial. *Trials.* 2021;22(1):148.

69. Falconer C, Park M, Skow A, et al. Scoping the impact of the national child measurement programme feedback on the child obesity pathway: study protocol. *BMC Public Health.* 2012;12:783.

70. Hill JL, Heelan KA, Bartee RT, et al. A Type III Hybrid Effectiveness-Implementation Pilot Trial Testing Dissemination and Implementation Strategies for a Pediatric Weight Management Intervention: The Nebraska Childhood Obesity Research Demonstration Project. *Child Obes.* 2021;17(S1):S70-s78.

71. Karmali S, Ng V, Battram D, et al. Coaching and/or education intervention for parents with overweight/obesity and their children: study protocol of a single-centre randomized controlled trial. *BMC Public Health.* 2019;19(1):345.

72. Kim JE, Jang HB, Lee HJ, et al. Rationale and design of theory-based nutritional strategy to promote healthy dietary habits in severe obese adolescents: Study protocol for ICAAN (intervention for childhood and adolescent obesity via activity and nutrition) project. *FASEB Journal.* 2017;31(1).

73. Lim C, Rutledge L, Sandridge S, King K, Jefferson D, Tucker T. Design, Implementation, and Examination of a Remote Patient Monitoring System for Pediatric Obesity: Protocol for an Open Trial Pilot Study. *JMIR Res Protoc.* 2021;10(7):e29858.

74. Liu S, Marques IG, Perdew MA, et al. Family-based, healthy living intervention for children with overweight and obesity and their families: a 'real world' trial protocol using a randomised wait list control design. *BMJ Open.* 2019;9(10):e027183.

75. Mâsse LC, Vlaar J, Macdonald J, et al. Aim2Be mHealth intervention for children with overweight and obesity: study protocol for a randomized controlled trial. *Trials.* 2020;21(1):132.

76. Moores CJ, Maeder A, Miller J, et al. A Digital Intervention for Australian Adolescents Above a Healthy Weight (Health Online for Teens): Protocol for an Implementation and User Experience Study. *JMIR Res Protoc.* 2019;8(10):e13340.

77. Moorhead A, Coates V, Hazlett D, et al. Weight Care Project: Health professionals' attitudes and ability to assess body weight status - study protocol. *BMC public health.* 2011;11:202.

78. Morgan PJ, Collins CE, Plotnikoff RC, et al. The SHED-IT community trial study protocol: a randomised controlled trial of weight loss programs for overweight and obese men. *BMC public health.* 2010;10:701.

79. O'Malley G, Clarke M, Burls A, Murphy S, Murphy N, Perry IJ. A smartphone intervention for adolescent obesity: study protocol for a randomised controlled non-inferiority trial. *Trials.* 2014;15:43.

80. Parkinson KN, Jones AR, Tovee MJ, et al. A cluster randomised trial testing an intervention to improve parents' recognition of their child's weight status: study protocol. *BMC Public Health.* 2015;15:549.

81. Parretti HM, Ives NJ, Tearne S, et al. Protocol for the feasibility and acceptability of a brief routine weight management intervention for postnatal women embedded within the national child immunisation programme: randomised controlled cluster feasibility trial with nested qualitative study (PIMMS-WL). *BMJ Open.* 2020;10(2):e033027.

82. Pbert L, Trivedi M, Druker S, et al. Supporting families of children with overweight and obesity to live healthy lifestyles: Design and rationale for the Fitline cluster randomized controlled pediatric practice-based trial. *Contemp Clin Trials.* 2021;104:106348.

83. Ramalho S, Saint-Maurice PF, Silva D, et al. APOLO-Teens, a web-based intervention for treatment-seeking adolescents with overweight or obesity: study protocol and baseline characterization of a Portuguese sample. *Eat Weight Disord.* 2020;25(2):453-463.

84. Shrewsbury VA, O'Connor J, Steinbeck KS, et al. A randomised controlled trial of a community-based healthy lifestyle program for overweight and obese adolescents: the Loozit study protocol. *BMC Public Health.* 2009;9:119.

85. Timpel P, Cesena FHY, Costa CD, et al. Efficacy of gamification-based smartphone application for weight loss in overweight and obese adolescents: study protocol for a phase II randomized controlled trial. *THERAPEUTIC ADVANCES IN ENDOCRINOLOGY AND METABOLISM.* 2018;9(6):167-176.

86. Tragomalou A, Kassari P, Ioakeimidis I, et al. BigO: The use of new technologies for the management of childhood obesity-A clinical pilot study. *Hormone Research in Paediatrics.* 2019;91:544.

87. Tragomalou A, Kassari P, Ioakeimidis I, et al. BigO: Novel technologies for the management of childhood obesity-The case of a clinical study in Greece. *Obesity Reviews.* 2020;21(SUPPL 1).

88. Wake M, Lycett K, Sabin MA, et al. A shared-care model of obesity treatment for 3-10 year old children: protocol for the HopSCOTCH randomised controlled trial. *BMC Pediatr.* 2012;12:39.

89. Willeboordse M, van de Kant KD, de Laat MN, van Schayck OC, Mulkens S, Dompeling E. Multifactorial intervention for children with asthma and overweight (Mikado): study design of a randomised controlled trial. *BMC Public Health.* 2013;13:494.

90. Wright ME, Delacroix E, Sonneville KR, et al. Reducing paediatric overweight and obesity through motivational interviewing: study protocol for a randomised controlled trial in the AAP PROS research network. *BMJ Open.* 2020;10(7):e035720.

91. Ansari MR, Kodriati N, Pertiwi AAP, Dewi FST. The Effectiveness of a Telenutrition Intervention to Improve Dietary Behavior and Physical Activity Among Adolescents With Obesity: Protocol for a Systematic Review. *JMIR Res Protoc.* 2024;13:e53282.

92. Assemany CG, Cunha DB, Brandão JM, et al. A multicomponent family intervention, combined with salt reduction for children with obesity: a factorial randomized study protocol. *BMC Public Health.* 2023;23(1):1453.

93. Barlow SE, Yudkin J, Nelson V, Allicock MA. Dynamo Kids!/¡Niños Dinámicos! A Web Site for Pediatric Primary Care Providers to Offer Parents of Children 6-12 Years Old With Overweight and Obesity: Web Site Development and Protocol for Pilot Study. *J Pediatr Health Care.* 2023;37(1):17-24.

94. Bernhardsson S, Boman C, Lundqvist S, et al. Implementation of physical activity on prescription for children with obesity in paediatric health care (IMPA): protocol for a feasibility and evaluation study using quantitative and qualitative methods. *Pilot Feasibility Stud.* 2022;8(1):117.

95. Cunningham PB, Naar S, Roberts JR, et al. Study protocol for clinical trial of the FIT Families multicomponent obesity intervention for African American adolescents and their caregivers: Next step from the ORBIT initiative. *BMJ Open.* 2024;14(2):e074552.

96. Davis AM, Befort CA, Lancaster BD, et al. Rationale and design of integrating a parents first obesity intervention with a pediatric weight management intervention for rural families - Evaluating the ripple effect. *Contemp Clin Trials.* 2023;128:107140.

97. Neshteruk CD, Skinner AC, Counts J, et al. Translating knowledge into action for child obesity treatment in partnership with Parks and Recreation: study protocol for a hybrid type II trial. *Implement Sci.* 2023;18(1):6.

98. O'Reilly SL, Burden C, Campoy C, et al. Bump2Baby and Me: protocol for a randomised trial of mHealth coaching for healthy gestational weight gain and improved postnatal outcomes in high-risk women and their children. *Trials.* 2021;22(1):963.

99. Pare SM, Gunn E, Morrison KM, et al. Testing a Biobehavioral Model of Chronic Stress and Weight Gain in Young Children (Family Stress Study): Protocol and Baseline Demographics for a Prospective Observational Study. *JMIR Res Protoc.* 2024;13:e48549.

100. Tian C, Xu J, Wang G, Yu L, Tang X. The effectiveness of web-based interventions on non-alcoholic fatty liver disease (NAFLD) in obese children: A study protocol for a randomized controlled trial. *Front Public Health.* 2022;10:930901.

101. Zare Z, Hajizadeh E, Mahmoodi M, Nazari R, Shahmoradi L, Rezayi S. Smartphone-based application to control and prevent overweight and obesity in children: design and evaluation. *BMC Med Inform Decis Mak.* 2023;23(1):201.

102. Zhu D, Dordevic AL, Gibson S, Davidson ZE. Evaluating a 10-Week Family-Focused E-Health Healthy Lifestyle Program for School-Aged Children with Overweight or Obesity: A Randomized Controlled Trial Study Protocol. *Nutrients.* 2023;15(13).

103. Mani S, Joseph LH, Sharma S. Feasibility of telemedicine or telephone-based family intervention for rural paediatric obesity: Cluster randomized control trial. *J Telemed Telecare*. Jun 2016;22(4):264-5. doi:10.1177/1357633x15601524

104. Abraham AA, Chow WC, So HK, Woo J, Chan SM, Nelson EAS. Feasibility of Using Cell Phone Reminders to Motivate Behaviour Change in Obese Adolescents in Hong Kong. *Hong Kong Journal of Paediatrics.* 2014;19(3):197.

105. Chen JL, Guedes CM, Cooper BA, Lung AE. Short-Term Efficacy of an Innovative Mobile Phone Technology-Based Intervention for Weight Management for Overweight and Obese Adolescents: Pilot Study. *Interact J Med Res.* 2017;6(2):e12.

106. Chen JL, Guedes CM, Lung AE. Smartphone-based Healthy Weight Management Intervention for Chinese American Adolescents: Short-term Efficacy and Factors Associated With Decreased Weight. *J Adolesc Health.* 2019;64(4):443-449.

107. Davis AM, Sampilo M, Gallagher KS, et al. Treating rural paediatric obesity through telemedicine vs. telephone: Outcomes from a cluster randomized controlled trial. *J Telemed Telecare.* 2016;22(2):86-95.

108. Maddison R, Foley L, Ni Mhurchu C, et al. Effects of active video games on body composition: a randomized controlled trial. *Am J Clin Nutr.* 2011;94(1):156-163.

109. Taveras EM, Marshall R, Sharifi M, et al. Comparative Effectiveness of Clinical-Community Childhood Obesity Interventions: A Randomized Clinical Trial. *JAMA Pediatr.* 2017;171(8):e171325.

110. Tully L, Sorensen J, O'Malley G. Pediatric Weight Management Through mHealth Compared to Face-to-Face Care: Cost Analysis of a Randomized Control Trial. *JMIR Mhealth Uhealth.* 2021;9(9):e31621.

111. Wilson DK, Sweeney AM, Van Horn ML, et al. The Results of the Families Improving Together (FIT) for Weight Loss Randomized Trial in Overweight African American Adolescents. *Ann Behav Med.* 2022;56(10):1042-1055.

112. Zhang Q, O'Connor DB, Hugh-Jones S. Feasibility of a multiple-component mindfulness intervention for Chinese adolescents living with overweight: A pilot randomized trial. *Appl Psychol Health Well Being.* 2023;15(2):516-535.

113. Ajie WN, Chapman-Novakofski KM. Impact of computer-mediated, obesity-related nutrition education interventions for adolescents: a systematic review. *J Adolesc Health.* 2014;54(6):631-645.

114. Altman M, Wilfley DE. Evidence update on the treatment of overweight and obesity in children and adolescents. *J Clin Child Adolesc Psychol.* 2015;44(4):521-537.

115. An JY, Hayman LL, Park YS, Dusaj TK, Ayres CG. Web-based weight management programs for children and adolescents: a systematic review of randomized controlled trial studies. *ANS Adv Nurs Sci.* 2009;32(3):222-240.

116. Antwi F, Fazylova N, Garcon MC, Lopez L, Rubiano R, Slyer JT. The effectiveness of web-based programs on the reduction of childhood obesity in school-aged children: A systematic review. *JBI Libr Syst Rev.* 2012;10(42 Suppl):1-14.

117. Antwi FA, Fazylova N, Garcon MC, Lopez L, Rubiano R, Slyer JT. Effectiveness of web-based programs on the reduction of childhood obesity in school-aged children: A systematic review. *JBI Database of Systematic Reviews and Implementation Reports.* 2013;11(6):1-44.

118. Azevedo LB, Stephenson J, Ells L, et al. The effectiveness of e-health interventions for the treatment of overweight or obesity in children and adolescents: A systematic review and meta-analysis. *Obes Rev.* 2021:e13373.

119. Baker STE, Yucel M, Fornito A, Allen NB, Lubman DI. A systematic review of diffusion weighted MRI studies of white matter microstructure in adolescent substance users. *NEUROSCIENCE AND BIOBEHAVIORAL REVIEWS.* 2013;37(8):1713-1723.

120. Bernardi L, Novello D. Analysis of the influence of television on the levels of obesity in adolescents: a systematic review. *RBONE-REVISTA BRASILEIRA DE OBESIDADE NUTRICAO E EMAGRECIMENTO.* 2017;11(64):199-210.

121. Bryant MJ, Lucove JC, Evenson KR, Marshall S. Measurement of television viewing in children and adolescents: A systematic review. *Obesity Reviews.* 2007;8(3):197-209.

122. Chang TH, Chen YC, Chen WY, et al. Weight Gain Associated with COVID-19 Lockdown in Children and Adolescents: A Systematic Review and Meta-Analysis. *Nutrients.* 2021;13(10).

123. Chaplais E, Naughton G, Thivel D, Courteix D, Greene D. Smartphone Interventions for Weight Treatment and Behavioral Change in Pediatric Obesity: A Systematic Review. *Telemed J E Health.* 2015;21(10):822-830.

124. Cliff DP, Ridgers ND, Tsiros MD, et al. Associations between objectively measured sedentary behaviour and adiposity in children and adolescents: Systematic review and meta-analysis. *Journal of Science and Medicine in Sport.* 2014;18:e154.

125. DeSilva S, Vaidya SS. The Application of Telemedicine to Pediatric Obesity: Lessons from the Past Decade. *Telemed J E Health.* 2021;27(2):159-166.

126. Duriancik DM, Goff CR. Children of single-parent households are at a higher risk of obesity: A systematic review. *J Child Health Care.* 2019;23(3):358-369.

127. Faulkner GEJ, Buliung RN, Flora PK, Fusco C. Active school transport, physical activity levels and body weight of children and youth: A systematic review. *PREVENTIVE MEDICINE.* 2009;48(1):3-8.

128. Ferreira MS, Marson FAL, Wolf VLW, Ribeiro JD, Mendes RT. Lung function in obese children and adolescents without respiratory disease: a systematic review. *BMC Pulm Med.* 2020;20(1):281.

129. Ghobadi S, Hassanzadeh-Rostami Z, Salehi-Marzijarani M, et al. Association of eating while television viewing and overweight/obesity among children and adolescents: a systematic review and meta-analysis of observational studies. *Obes Rev.* 2018;19(3):313-320.

130. Hamel LM, Robbins LB, Wilbur J. Computer- and web-based interventions to increase preadolescent and adolescent physical activity: A systematic review. *Journal of Advanced Nursing.* 2011;67(2):251-268.

131. Hammersley ML, Jones RA, Okely AD. Parent-Focused Childhood and Adolescent Overweight and Obesity eHealth Interventions: A Systematic Review and Meta-Analysis. *J Med Internet Res.* 2016;18(7):e203.

132. Jesus GDS, Costa PRF, Oliveira LPM, et al. Body Adiposity and Apolipoproteins in Children and Adolescents: A Meta-Analysis of Prospective Studies. *Arq Bras Cardiol.* 2020;115(2):163-171.

133. Kaakinen P, Kyngäs H, Kääriäinen M. Technology-based counseling in the management of weight and lifestyles of obese or overweight children and adolescents: A descriptive systematic literature review. *Inform Health Soc Care.* 2018;43(2):126-141.

134. Kracht CL, Hutchesson M, Ahmed M, et al. E-&mHealth interventions targeting nutrition, physical activity, sedentary behavior, and/or obesity among children: A scoping review of systematic reviews and meta-analyses. *OBESITY REVIEWS.* 2021;22(12).

135. Lampe EW, Abber SR, Forman EM, Manasse SM. Guidelines for caregivers and healthcare professionals on speaking to children about overweight and obesity: a systematic review of the gray literature. *Transl Behav Med.* 2020;10(5):1144-1154.

136. Langarizadeh M, Sadeghi M, As'habi A, Rahmati P, Sheikhtaheri A. Mobile apps for weight management in children and adolescents; An updated systematic review. *Patient Educ Couns.* 2021;104(9):2181-2188.

137. Lee J, Piao M, Byun A, Kim J. A Systematic Review and Meta-Analysis of Intervention for Pediatric Obesity Using Mobile Technology. *Studies in health technology and informatics.* 2016;225:491-494.

138. Margetin CA, Rigassio Radler D, Thompson K, et al. Anthropometric Outcomes of Children and Adolescents Using Telehealth with Weight Management Interventions Compared to Usual Care: A Systematic Review and Meta-analysis. *J Am Coll Nutr.* 2021:1-24.

139. Marker C, Gnambs T, Appel M. Exploring the myth of the chubby gamer: A meta-analysis on sedentary video gaming and body mass. *Social Science and Medicine.* 2019.

140. Masoumi HE. ACTIVE TRANSPORT TO SCHOOL AND CHILDREN'S BODY WEIGHT A SYSTEMATIC REVIEW. *TEMA-JOURNAL OF LAND USE MOBILITY AND ENVIRONMENT.* 2017;10(1):95-110.

141. McGavock J, Chauhan BF, Rabbani R, et al. Layperson-Led vs Professional-Led Behavioral Interventions for Weight Loss in Pediatric Obesity: A Systematic Review and Meta-analysis. *JAMA Netw Open.* 2020;3(7):e2010364.

142. McMullan M, Millar R, Woodside JV. A systematic review to assess the effectiveness of technology-based interventions to address obesity in children. *BMC Pediatr.* 2020;20(1):242.

143. Meidani Z, Nabovati E, Gohari S, Chopannejad S. Phone-based interventions to control obesity in children under six years of age: A systematic review on features and effects. *Journal of Comprehensive Pediatrics.* 2018;9(3).

144. Mitchell TB, Amaro CM, Steele RG. Pediatric Weight Management Interventions in Primary Care Settings: A Meta-Analysis. *Health Psychol.* 2016.

145. Moorman EL, Koskela-Staples NC, Mathai BB, Fedele DA, Janicke DM. Pediatric Obesity Treatment via Telehealth: Current Evidence and Future Directions. *Curr Obes Rep.* 2021;10(3):371-384.

146. Neves MEA, de Souza MR, Gorgulho BM, Cunha DB, Muraro AP, Rodrigues PRM. Association of dietary patterns with blood pressure and body adiposity in adolescents: a systematic review. *EUROPEAN JOURNAL OF CLINICAL NUTRITION.* 2021;75(10):1440-1453.

147. O'Brien A. "Web-based weight management programs for children and adolescents: a systematic review of randomized controlled trial studies" by An, Hayman, Park, Dusaj, and Ayres (July-September 2009, Vol 32, No 3, pp 222-240). *ANS Adv Nurs Sci.* 2010;33(1):2.

148. Perego P, Rashid R, Gluud C, Jakobsen JC, Andreoni G, Lissau I. Comparison of different mobile health applications for intervention in children and adolescent with overweight: a protocol for systematic review with meta-analysis and trial sequential analysis. *BMJ Open.* 2020;10(12):e032570.

149. Pourmoradian S, Ostadrahimi A, Bonab AM, Roudsari AH, Jabbari M, Irandoost P. Television food advertisements and childhood obesity: A systematic review. *International Journal for Vitamin and Nutrition Research.* 2021;91(1-2):3-9.

150. Rashid R, Perego P, Condon L, et al. Health apps targeting children with overweight-a protocol for a systematic review with meta-analysis and Trial Sequential Analysis of randomised clinical trials. *Syst Rev.* 2020;9(1):28.

151. Reddy P, Dukhi N, Sewpaul R, Ellahebokus MAA, Kambaran NS, Jobe W. Mobile Health Interventions Addressing Childhood and Adolescent Obesity in Sub-Saharan Africa and Europe: Current Landscape and Potential for Future Research. *Front Public Health.* 2021;9:604439.

152. Rossi CE, Albernaz DO, de Vasconcelos FDG, de Assis MAA, Di Pietro PF. Television influence on food intake and obesity in children and adolescents: a systematic review. *REVISTA DE NUTRICAO-BRAZILIAN JOURNAL OF NUTRITION.* 2010;23(4):607-620.

153. Santos REA, Lacerda DC, da Silva MG, Barbosa DAM, Pinheiro IL, Pereira KNF. Mastication in children and adolescents with overweight or obesity: a systematic review. *REVISTA DE NUTRICAO-BRAZILIAN JOURNAL OF NUTRITION.* 2021;34.

154. Sonntag D, Schneider S, Mdege N, Ali S, Schmidt B. Beyond Food Promotion: A Systematic Review on the Influence of the Food Industry on Obesity-Related Dietary Behaviour among Children. *Nutrients.* 2015;7(10):8565-8576.

155. Te Velde SJ, Van Nassau F, Uijtdewilligen L, et al. Energy balance-related behaviours associated with overweight and obesity in preschool children: A systematic review of prospective studies. *Obesity Reviews.* 2012;13(SUPPL. 1):56-74.

156. Vescio A, Testa G, Montemagno M, Sapienza M, Pavone V. Secondary displacement risk after reduction and cast immobilization of displaced distal radius fractures in overweight and obese children: a systematic review and meta-analysis. *MINERVA ORTHOPEDICS.* 2021;72(1):71-76.

157. Whitley A, Yahia N. Efficacy of Clinic-Based Telehealth vs. Face-to-Face Interventions for Obesity Treatment in Children and Adolescents in the United States and Canada: A Systematic Review. *Child Obes.* 2021;17(5):299-310.

158. Wickham CA, Carbone ET. Who's calling for weight loss? A systematic review of mobile phone weight loss programs for adolescents. *Nutr Rev.* 2015;73(6):386-398.

159. Azevedo LB, Stephenson J, Ells L, et al. The effectiveness of e-health interventions for the treatment of overweight or obesity in children and adolescents: A systematic review and meta-analysis. *Obes Rev.* 2022;23(2):e13373.

160. Bleiweiss-Sande R, Skelton K, Zaltz D, Bacardí-Gascón M, Jiménez-Cruz A, Benjamin-Neelon SE. Interventions to prevent obesity in Latinx children birth to 6 years globally: a systematic review. *Public Health Nutr.* 2023;26(11):2498-2513.

161. Chai LK, Farletti R, Fathi L, Littlewood R. A Rapid Review of the Impact of Family-Based Digital Interventions for Obesity Prevention and Treatment on Obesity-Related Outcomes in Primary School-Aged Children. *Nutrients.* 2022;14(22).

162. Davison GM, Monocello LT, Lipsey K, Wilfley DE. Evidence Base Update on Behavioral Treatments for Overweight and Obesity in Children and Adolescents. *J Clin Child Adolesc Psychol.* 2023;52(5):589-603.

163. Hassapidou M, Duncanson K, Shrewsbury V, et al. EASO and EFAD Position Statement on Medical Nutrition Therapy for the Management of Overweight and Obesity in Children and Adolescents. *Obes Facts.* 2023;16(1):29-52.

164. Henes ST, Stotz SA, Riggs SE, Yang HM. eHealth, family-based interventions, and multilevel approaches to pediatric weight management: a scoping review. *Nutr Rev.* 2024.

165. Kirk S, Ogata B, Wichert E, Handu D, Rozga M. Treatment of Pediatric Overweight and Obesity: Position of the Academy of Nutrition and Dietetics Based on an Umbrella Review of Systematic Reviews. *J Acad Nutr Diet.* 2022;122(4):848-861.

166. Kouvari M, Karipidou M, Tsiampalis T, et al. Digital Health Interventions for Weight Management in Children and Adolescents: Systematic Review and Meta-analysis. *J Med Internet Res.* 2022;24(2):e30675.

167. Lin C, Li D, Wang X, Yang S. Chronic exercise interventions for executive function in overweight children: a systematic review and meta-analysis. *Front Sports Act Living.* 2024;6:1336648.

168. Margetin CA, Rigassio Radler D, Thompson K, et al. Anthropometric Outcomes of Children and Adolescents Using Telehealth with Weight Management Interventions Compared to Usual Care: A Systematic Review and Meta-analysis. *J Am Nutr Assoc.* 2022;41(2):207-229.

169. Metzendorf MI, Wieland LS, Richter B. Mobile health (m-health) smartphone interventions for adolescents and adults with overweight or obesity. *Cochrane Database Syst Rev.* 2024;2(2):Cd013591.

170. Mirza M, Brown-Hollie JP, Suarez-Balcazar Y, et al. Interventions for Health Promotion and Obesity Prevention for Children and Adolescents with Developmental Disabilities: a Systematic Review. *Rev J Autism Dev Disord.* 2022:1-24.

171. O'Hara VM, Louder D, Johnston SV, Hastey K, Browne NT. Pediatric Obesity Care via Telemedicine: Expanding the Path Forward-A Review. *Curr Obes Rep.* 2023;12(4):546-556.

172. Pawellek S, Ziegeldorf A, Wulff H. [Strategies and effects of digital interventions in overweight and obesity treatments in children and adolescents-a systematic review]. *Bundesgesundheitsblatt Gesundheitsforschung Gesundheitsschutz.* 2022;65(5):624-634.

173. Soltero EG, Lopez C, Hernandez E, O'Connor TM, Thompson D. Technology-Based Obesity Prevention Interventions Among Hispanic Adolescents in the United States: Scoping Review. *JMIR Pediatr Parent.* 2022;5(4):e39261.

174. Vandoni M, Codella R, Pippi R, et al. Combatting Sedentary Behaviors by Delivering Remote Physical Exercise in Children and Adolescents with Obesity in the COVID-19 Era: A Narrative Review. *Nutrients.* 2021;13(12).

175. Yau KW, Tang TS, Görges M, et al. Effectiveness of Mobile Apps in Promoting Healthy Behavior Changes and Preventing Obesity in Children: Systematic Review. *JMIR Pediatr Parent.* 2022;5(1):e34967.

176. Zhang P, Tang X, Peng X, Hao G, Luo S, Liang X. Effect of screen time intervention on obesity among children and adolescent: A meta-analysis of randomized controlled studies. *Prev Med.* 2022;157:107014.

177. Zuñiga Vinueza AM, Jaramillo AP. The Effectiveness of a Healthy Lifestyle in Obese Pediatric Patients: A Systematic Review and Meta-Analysis. *Cureus.* 2023;15(11):e48525.
